# First records of tick-borne pathogens in populations of the taiga tick *Ixodes persulcatus* in Sweden

**DOI:** 10.1186/s13071-019-3813-0

**Published:** 2019-11-28

**Authors:** Thomas G. T. Jaenson, Peter Wilhelmsson

**Affiliations:** 1 Medical Entomology Unit, Department of Organismal Biology, Evolutionary Biology Centre, Uppsala University, Uppsala, Sweden; 2 Division of Medical Microbiology, Department of Clinical and Experimental Medicine, Linköping University, Linköping, Sweden; 3 Department of Clinical Microbiology, Region Jönköping County, Jönköping, Sweden

**Keywords:** Taiga tick, Sweden, *Borrelia afzelii*, *B. garinii*, *B. valaisiana*, *Rickettsia helvetica*, *Ixodes persulcatus*, Geese

## Abstract

**Background:**

The common tick *Ixodes ricinus* and the taiga tick *I. persulcatus* are the main tick vectors of *Borrelia* spirochaetes, TBE virus (TBEV) and of several other zoonotic pathogens in the western and eastern areas, respectively of the Palaearctic region. Recently, populations of the taiga tick were, for the first time, detected in northern Sweden. This prompted us to investigate if they harbour human pathogens.

**Methods:**

A total of 276 *I. persulcatus* ticks (136 males, 126 females and 14 nymphs) and one *I. ricinus* nymph was collected by the cloth-dragging method in northern Sweden in July–August 2015 and May–July 2016. In addition, 8 males and 10 females of *I. persulcatus* were collected from two dogs (16 and 2 ticks, respectively) in two of the localities. All ticks were microscopically and molecularly identified to developmental stage and species and screened for *B. burgdorferi* (*sensu lato*), *B. miyamotoi*, *Anaplasma phagocytophilum*, *Rickettsia* spp.*, Neoehrlichia mikurensis*, *Babesia* spp. and TBEV using real-time PCR followed by species identification by sequencing the PCR-products of conventional PCR assays.

**Results:**

Of the ticks collected by the cloth-dragging method, 55% (152/277) were positive for *Borrelia*. There was no significant difference between the proportions of *Borrelia*-infected nymphs (33%, 5/15) and *Borrelia*-infected adult ticks (56%, 147/262), and no significant difference between the proportions of *Borrelia*-infected males (54%, 74/136) and *Borrelia*-infected females (58%, 73/126). Three different *Borrelia* species were identified. *Borrelia afzelii* was the predominant species and detected in 46% of all *Borrelia*-infected ticks followed by *B. garinii*, 35%, *B. valaisiana*, 1%, and mixed infections of different *Borrelia* species, 1%; 17% of all *Borrelia*-infections were untypeable. One *I. persulcatus* female contained *Rickettsia helvetica*, and one nymph contained *Rickettsia* sp. Of the 277 ticks analysed, all were negative for *A. phagocytophilum*, *Babesia* spp., *Borrelia miyamotoi*, *N. mikurensis* and TBEV. The ticks collected from the two dogs were negative for all pathogens examined except for *Borrelia* spp., that was detected in 5 out of 16 ticks removed from one of the dogs.

**Conclusions:**

To our knowledge, this is the first time that *I. persulcatus* from Sweden has been analysed for the presence of tick-borne pathogens. The examined tick populations had a low diversity of tick-borne pathogens but a high prevalence of *B. burgdorferi* (*s.l*.).

## Background

Two closely related tick species, *Ixodes ricinus* and *I. persulcatus*, are the main vectors of Lyme borreliosis spirochaetes, of the tick-borne encephalitis virus (TBEV) and of several other tick-borne pathogens of humans in the western and eastern Palaearctic region, respectively. In Sweden, the common tick *I. ricinus* is the main vector of tick-borne pathogens to humans [1, 2]. It is abundant in the southern and south-central parts of the country. In inland northern Sweden and along the northern east coast bordering the Baltic Sea the distribution of *I. ricinus* is more scattered [1, 2]. Recently in 2015, permanent populations of the taiga tick, *I. persulcatus* were detected for the first time in northern Sweden [3]. Until then, its known geographical distribution was from eastern Latvia and Estonia, and Finland eastwards through Russia into Mongolia and parts of the Peoples Republic of China, Taiwan and North Korea to Japan [4, 5]. The vegetation type usually inhabited by *I. persulcatus* is mixed deciduous–coniferous forest [4, 5].

Several proven or putative pathogens of humans have been recorded from the taiga tick. They include *Borrelia miyamotoi*, *B. burgdorferi* (*sensu stricto*), *B. afzelii*, *B. garinii*, *B. bavariensis*, *Rickettsia heilongjiangensis*, *R. helvetica*, *R. raoultii*, *R. sibirica*, “*Candidatus* Rickettsia tarasevichiae”, *Anaplasma phagocytophilum*, *Ehrlichia muris*, *Neoehrlichia mikurensis*, *Bartonella henselae*, *Babesia divergens*, *Ba. microti*, *Ba. venatorum*, tick-borne encephalitis virus (TBEV) and Kemerovo virus [6–20].

Lyme borreliosis (LB) is the most common tick-borne disease in the temperate regions of Europe, North America and Asia; 65,000 human cases of LB are estimated to occur annually in Europe [21]. LB is prevalent also in Asia [13]; and in the USA there are presumably > 300,000 human cases of LB each year [22]. However, the potentially most severe and most important of the *Ixodes*-vectored pathogens in the Palaearctic region is the TBEV, which during 1990–2009 caused between 6000 and 12,000 human cases annually in Europe including Russia [9].

This prompted us to investigate the potential occurrence of the TBEV, different genospecies of *B. burgdorferi* (*s.l*.), *Borrelia miyamotoi*, *Rickettsia* spp., *Anaplasma phagocytophilum*, *N. mikurensis* and *Babesia* spp. in the recently discovered populations of the taiga tick in northern Sweden. The main aim was to analyse the potential medical importance of *I. persulcatus* in the study area.

## Methods

### Climate and vegetation

As described by Jaenson et al. [3], the locations from where we collected ticks are situated in the Bothnian Bay area, province of Norrbotten in northern Sweden. The region belongs to the middle boreal subzone [23]. Norrbotten is to the west bordered by the Swedish province Lappland, which together with Norrbotten form the northernmost provinces of Sweden. This is a typical taiga region, strongly dominated by forests but also in part by mires. It reaches in altitude to about to 150–200 m a.s.l. in Norrbotten. In the Bothnian Bay area, where we found *I. persulcatus* at several localities, the length of the vegetation period is about 140–150 days and the annual precipitation 600–700 mm. The July mean temperature is 15–16 °C. The human population density is low compared with southern Sweden. Most of the 500,000 inhabitants of the Bothnian Bay area reside in the coastal regions and the river valleys.

### Tick collection localities

**Fig. 1:**
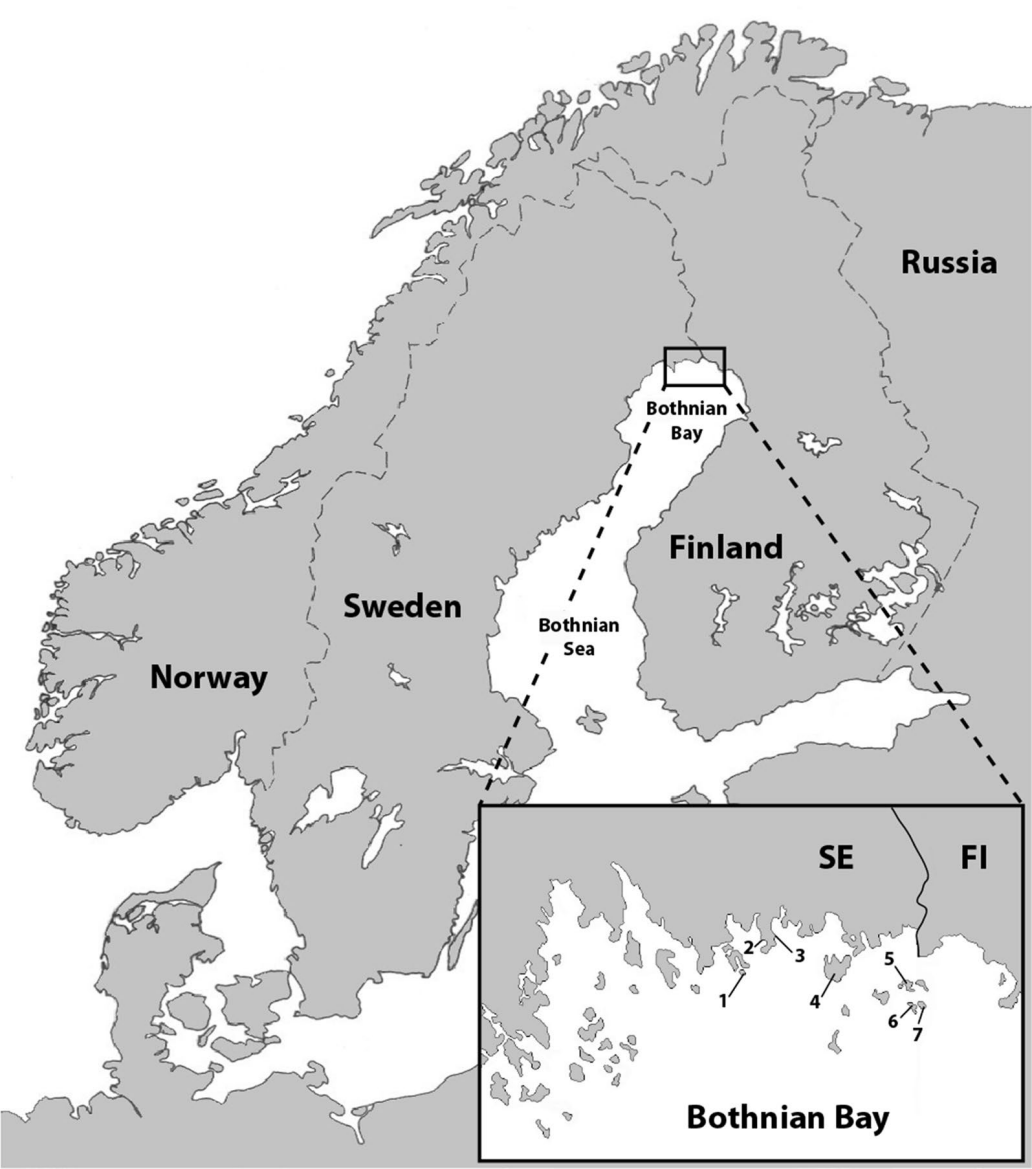
Map showing the seven localities where ticks were collected by cloth-dragging method. Ticks [adult males (AM); adult females (AF); nymphs (N)] were collected in the Bothnian Bay area, province of Norrbotten, northern Sweden in July-August 2015 and May-July 2016. Numbers (1–7) correspond to the following localities: 1, Ytterstlandet (AM = 114; AF = 108; N = 4); 2, Axelsvik (AM = 1; AF = 1; N = 1); 3, Båtskärsnäs (AM = 2); 4, Seskarö (AF = 1); 5. Stora Hamnskär (AM = 1); 6, Västra Knivskär (AM = 18; AF = 16; N = 8); 7, Östra Knivskär (N = 2)

We contacted dog-owners and hunters living in the coastal areas of the provinces of northern Sweden and asked if they and/or their dogs had become infested by ticks in the Baltic Bay area. We then asked for the name(s) of the presumed locality/localities where any tick infestation had occurred. Based on this information we searched for ticks at a total of 49 localities in the Bothnian Bay area, province of Norrbotten, northern Sweden in July–August 2015 and May–July 2016. Ticks were detected by the cloth-dragging method at seven mixed woodland, island localities in the Bothnian Bay, province of Norrbotten, northern Sweden (Fig. 1): Axelsvik (65°46′31″N, 23°23′11″E); Båtskärsnäs (65°47′05.88″N, 23°25′13.85″E); Seskarö (65°42′20″N, 23°43′30″E); Stora Hamnskär (65°42′21.45″N, 24°6′35.75″E and 65°42′21,50″N, 24°6′27,92″E); Västra Knivskär (65°4′33″N, 24°6′46″E); Östra Knivskär (65°40′3″N, 24°8′59″E and 65°40′33″N, 24°8′54″E); and Ytterstlandet (65°42′34″N, 23°17′20″E).

### Tick collection

All sampling occasions took place during daytime when the ground vegetation was not wet due to recent or ongoing rainfall. At each sampling locality a white woolen 1 m^2^ cloth was pulled at slow walking pace over the ground vegetation and/or field vegetation for a total of 300 m. At every 10 m of dragging, a stop was done and both sides of the cloth were inspected with the help of a magnifying lens. Any tick detected was put in a numbered plastic vial containing 80% ethanol. A total number of 277 ticks was collected by the cloth-dragging method. In addition, 18 ticks were removed and collected from two dogs at Ytterstlandet (*n* = 16), and Stora Hamnskär (*n* = 2), respectively.

### Tick identification

All tick specimens were identified to stage and species by morphological characteristics as described in more detail in [3]. A Leitz Wild M10 stereomicroscope was used together with keys and illustrations in [4, 5] and [24].

### Nucleic acid extraction and cDNA synthesis from ticks

Collected ticks were homogenized individually by bead-beating in 2 ml safe-lock microcentrifuge tubes (Eppendorf AG, Hamburg, Germany) with a 5-mm stainless steel bead (Qiagen, Hilden, Germany) in 350 µl RLT buffer (Qiagen), supplemented with 1% 2-mercaptoethanol (Sigma-Aldrich, Stockholm, Sweden), using a TissueLyser II (Qiagen) for 2 min at 25 Hz. After centrifugation at 20,000× *g* for 3 min, 300 µl supernatant were transferred to new microcentrifuge tubes for total nucleic acid (NA) extraction, using MagAttract^®^ Viral RNA M48 kit (Qiagen) in a BioRobot M48 workstation (Qiagen), using a 65-µl elution volume. Each batch of 24 samples consisted of 22 ticks, one positive control [5 µl of *B. burgdorferi* (*sensu stricto*) B31 ATCC 35210 (10^8^ cells/ml), and 5 µl inactivated TBEV strain K23, Encepur^®^, Chiron Vaccines, Marburg, Germany] and one negative control (H_2_O) that were extracted simultaneously.

The eluted NA was reverse-transcribed to cDNA using illustra™ Ready-to-Go RT-PCR Beads kit (GE Healthcare, Amersham Place, UK). Twenty microliters NA and 10 µl pd(N)6 random hexamer primers (0.25 µg/µl) were incubated for 5 min at 97 °C and then mixed with one RT-PCR bead dissolved in 20 µl RNase-free water. The mixture was incubated for 30 min at 42 °C, followed by 5 min at 97 °C, producing 50 µl cDNA.

### Molecular identification of tick species

Morphological identification of tick species was confirmed by molecular identification, using a species-specific duplex TaqMan real-time PCR assay, as previously described [25]. The primers IXO-I2-F4 and IXO-I2-R4 are designed to target the *Ixodes* spp. internal transcribed spacer (ITS2) to amplify genus-specific segments. The species-specific probes Iri-I2-P4 and Ipe-I2-P4 are designed to target either of the tick species (*I. ricinus* or *I. persulcatus*, respectively) (Table 1).

**Table 1:** Primers and probes used for molecular analysis of tick-borne pathogens and *Ixodes* ticks

Organism	Target	Primer/Probe name	Sequence (5′–3′)	Amplicon length (bp)	References
*A. phagocytophilum*	*gltA*	ApF	TTTTGGGCGCTGAATACGAT	64	Henningsson et al. [33]
		ApR	TCTCGAGGGAATGATCTAATAACGT		
		ApM	FAM-TGCCTGAACAAGTTATG-BHQ1		
*Babesia* spp.	*18S* rRNA	BJ1	GTCTTGTAATTGGAATGATGG	411–452	Casati et al. [39]
		BN2	TAGTTTATGGTTAGGACTACG		
*Borrelia* spp.	*16S* rRNA	B16S_FL	GAGTCGTCAAGACTGACGCTAAGTC	131	Wilhelmsson et al. [26]
		B16S_R	GCACACTTAACACGTTAGCTTCGGTACTAAC		
	5S–23S rRNA IGS	B5S-23S_F	CTGCGAGTTCGCGGGAGA	225–266	Postic et al. [27]
		B5S-23S_R	TCCTAGGCATTCACCATA		
		B5S-23S_Fn	GAGTTCGCGGGAGAGTAA		Wilhelmsson et al. [26]
		B5S-23S_Rn	TAGGCATTCACCATAGACTCTT		
*Borrelia miyamotoi*	*flaB*	Bm_F	AGAAGGTGCTCAAGCAG	156	Hovius et al. [28]
		Bm_R	TCGATCTTTGAAAGTGACATAT		
		Bm_P	FAM-AGCACAACAGGAGGGAGTTCAAGC-BHQ1		
*N. mikurensis*	*16S* rRNA	Neo_16S_F	GTAAAGGGCATGTAGGCGGTTTAA	107	Labbé Sandelin et al. [32]
		Neo_16S_R	TCCACTATCCTCTCTCGATCTCTAGTTTAA		
*Rickettsia* spp.	*gltA*	CS-F	TCGCAAATGTTCACGGTACTTT	74	Stenos et al. [34]
		CS-R	TCGTGCATTTCTTTCCATTGTG		
		CS-P	FAM-TGCAATAGCAAGAACCGTAGGCTGGATG-BHQ1		
	*17kDA*	Rr17kDa.61p	GCTCTTGCAACTTCTATGTT	434	Carl et al. [35]
		Rr17kDa.492n	CATTGTTCGTCAGGTTGGCG		
	*ompB*	Rc.rompB.4362p	GTCAGCGTTACTTCTTCGATGC	475	Choi et al. [36]
		Rc.rompB.4,836n	CCGTACTCCATCTTAGCATCAG		
		Rc.rompB.4,496p	CCAATGGCAGGACTTAGCTACT	267	
		Rc.rompB.4,762n	AGGCTGGCTGATACACGGAGTAA		
	*gltA*	RH314	AAACAGGTTGCTCATCATTC	837	Wallménius et al. [37]
		CSF-R	AAGTACCGTGAACATTTGCGA		
		CS-Ric-R	CAGTGAACATTTGCGACGGTA	832	
		CS535d	GCAATGTCTTATAAATATTC	Sequencing primer	
TBEV	11,054–11,121^a^	F-TBE 1	GGGCGGTTCTTGTTCTCC	68	Schwaiger & Cassinotti [30]
		R-TBE 1	ACACATCACCTCCTTGTCAGACT		
		TBE-probe-WT	FAM*-*TGAGCCACCATCACCCAGACACA-BHQ1		
	1329–1416^a^	TBEE-F6	GGCTTGTGAGGCAAAAAAGAA	88	Gäumann et al. [31]
		TBEE-R2	TCCCGTGTGTGGTTCGACTT		
		TBEE-P4	HEX*-*AAGCCACAGGACATGTGTACGACGCC-BHQ1		
*Ixodes* sp.	ITS2	IXO-I2-F4	TCTCGTGGCGTTGATTTGC	95	Sormunen et al. [25]
		IXO-I2-R4	CTGACGGAAGGCTACGACG		
		Ipe-I2-P4	FAM-TGCGTGGAAAGAAAACGAG-BHQ1		
		Iri-I2-P4	VIC-TGCTCGAAGGAGAGAACGA-BHQ1		

^a^Genome region of TBEV strain Neudoerfl (U27495)

*Abbreviations*: FAM, 6-carboxy-fluorescine; HEX, 6-carboxy-hexachlorofluoriscine; VIC, 6-carboxy-rhodamine; BHQ, Black Hole Quencher; IGS, intergenic spacer; ITS, internal transcribed spacer

A 20-µl reaction consisted of 10 µl Maxima^®^ Probe qPCR Master Mix (2X) (Thermo Fisher Scientific, Stockholm, Sweden), 0.4 µl of each primer (10 µM; Invitrogen; Table 1), 0.3 µl of probe Iri-I2-P4 (10 µM; Invitrogen; Table 1), 0.2 µl of probe Ipe-I2-P4 (10 µM; Invitrogen; Table 1), 6.7 µl RNase-free water and 2 µl cDNA template. The PCR reactions were performed on a C1000™ Thermal Cycler, CFX96^™^ Real-Time PCR Detection System (Bio-Rad Laboratories, Inc., Hercules, CA) using an activation step at 95 °C for 5 min, and 45 cycles of 95 °C for 10 s and 60 °C for 1 min.

### Detection of *Borrelia burgdorferi* (*sensu lato*) and determination of species

Detection of *Borrelia burgdorferi* (*sensu lato*) was done using a genus-specific LUX real-time PCR assay, as previously described [26]. The primers B16S_FL and B16S_R are designed to target the *Borrelia* spp. *16S* rRNA gene to amplify a 131 bp long amplicon (Table 1).

A 20-µl reaction consisted of 10 µl Platinum qPCR SuperMix uracil-D-glycosylase (UDG) (Invitrogen), 0.4 µl of each primer (10 µM; Invitrogen; Table 1), 7.2 µl RNase-free water and 2 µl cDNA template. The PCR reactions were performed on a C1000™ Thermal Cycler, CFX96™ Real-Time PCR Detection System (Bio-Rad Laboratories, Inc.) using an activation step at 50 °C for 2 min followed by 95 °C for 2 min, and then subjected to 45 cycles of 95 °C for 15 s, 58 °C for 30 s, and 72 °C for 30 s. Immediately after completion of PCR, melting curve analyses were performed by heating to 95 °C for 15 s, followed by cooling to 60 °C for 1 min, and subsequent heating to 95 °C at 0.8 °C min^−1^ with continuous fluorescence recording.

To determine *Borrelia burgdorferi* (*s.l.*) species of the samples positive in the LUX real-time PCR assay, a nested, conventional PCR assay using primers targeting the intergenic spacer region (IGS) between *5S* and *23S* rRNA genes (Table 1), was applied as previously described [26, 27]. Samples that failed to produce PCR products with this assay were denoted as ‘untypeable’.

### Detection of *Borrelia miyamotoi*

Detection of *B. miyamotoi* was done using a species-specific TaqMan real-time PCR assay, as previously described [28]. The primers Bm_F and Bm_R, and the probe Bm_P are designed to target the *B. miyamotoi* flagellin B gene (*flaB*) to amplify a 156-bp long amplicon (Table 1).

A 20-µl reaction consisted of 10 µl Maxima^®^ Probe qPCR Master Mix (2X) (Thermo Fisher Scientific), 0.4 µl of each primer (10 µM; Invitrogen; Table 1), 0.4 µl of probe (10 µM; Life Technologies; Table 1), 3.8 µl RNase-free water and 5 µl cDNA template. The PCR reactions were performed on a C1000™ Thermal Cycler, CFX96™ Real-Time PCR Detection System (Bio-Rad Laboratories, Inc.) using an activation step at 95 °C for 10 min, and 45 cycles of 95 °C for 5 s and 60 °C for 35 s, and finally one cycle of 37 °C for 20 s. As a positive control, a synthetic plasmid containing the target sequence of the TaqMan real-time PCR assay was used. The plasmid contained the target sequence, spanning the nucleotides 510–665 of the *B. miyamotoi* flagellin (*flaB*) gene (GenBank: KT932823), synthesized and cloned into Eurofins standard vector carrying the ampicillin selection marker (Eurofins Genomics, Ebersberg, Germany).

### Detection of TBEV

Detection of TBEV was done using a duplex TaqMan real-time PCR assay, as previously described [29]. The primers and probes are designed to target all three subtypes of TBEV to amplify a 68-bp and a 88-bp long amplicon, respectively [30, 31] (Table 1).

A 20-µl reaction consisted of 10 µl Maxima^®^ Probe qPCR Master Mix (2X) (Thermo Scientific™), 0.4 µl of each primer and probe (10 µM; Invitrogen; Table 1), 5.6 µl RNase-free water and 2 µl cDNA template. The PCR reactions were performed on a C1000™ Thermal Cycler, CFX96™ Real-Time PCR Detection System (Bio-Rad Laboratories, Inc.) using an activation step at 95 °C for 5 min, and 45 cycles of 95 °C for 10 s and 60 °C for 1 min.

### Detection of *Neoehrlichia mikurensis*

Detection of *N. mikurensis* was done using a SYBR green real-time PCR assay, as previously described [32]. The primers Neo_16S_F and Neo_16S_R are designed to target the *N. mikurensis 16S* rRNA gene to amplify a 107-bp long amplicon (Table 1).

A 20-µl reaction consisted of 10 µl SYBR™ Green PCR Master Mix (Thermo Fisher Scientific), 0.4 µl of each primer (10 µM; Invitrogen; Table 1), 7.2 µl RNase-free water and 2 µl cDNA template. The PCR reactions were performed on a C1000™ Thermal Cycler, CFX96™ Real-Time PCR Detection System (Bio-Rad Laboratories, Inc.) using an activation step at 95 °C for 3 min, and 45 cycles of 95 °C for 15 s, 60 °C for 30 s, and 72 °C for 30 s. Immediately after completion of PCR, melting curve analyses were performed by heating to 95 °C for 15 s, followed by cooling to 60 °C for 1 min, and subsequent heating to 95 °C at 0.8 °C min^-1^ with continuous fluorescence recording. As a positive control, cDNA samples positive for *N. mikurensis* confirmed by sequencing in an earlier study [32] were used in each run.

### Detection of *Anaplasma phagocytophilum*

Detection of *A. phagocytophilum* was done using a TaqMan real-time PCR assay, as previously described [33]. The primers ApF and ApR, and the probe ApM are designed to target the *A. phagocytophilum* citrate synthase gene (*gltA*) to amplify a 64-bp long amplicon (Table 1).

A 25-µl reaction consisted of 12.5 µl of 10 µl Maxima^®^ Probe qPCR Master Mix (2×) (Thermo Fisher Scientific), 1.5 µl of each primer and 0.375 µl of probe (10 µM; Invitrogen; Table 1), 7.125 µl RNase-free water and 2 µl cDNA template. The PCR reactions were performed on a C1000™ Thermal Cycler, CFX96™ Real-Time PCR Detection System (Bio-Rad Laboratories, Inc.) using an activation step at 95 °C for 10 min, and 40 cycles of 95 °C for 15 s and 60 °C for 1 min. As a positive control, a synthetic plasmid containing the target sequence of the TaqMan real-time PCR assay was used. The plasmid contained the target sequence, spanning the nucleotides 304–420 of the *A. phagocytophilum gltA* gene (GenBank: AF304137), synthesized and cloned into pUC57 vector (GenScript, Piscataway, NJ, USA).

### Detection of *Rickettsia* and determination of species

Detection of *Rickettsia* spp. was done using a TaqMan real-time PCR assay, as previously described [34]. The primers CS-F and CS-R, and probe CS-P are designed to target the *Rickettsia* spp. citrate synthase gene (*gltA*) to amplify a 74-bp long amplicon (Table 1).

A 20-µl reaction consisted of 10 µl Maxima^®^ Probe qPCR Master Mix (2×) (Thermo Fisher Scientific), 0.4 µl of each primer and probe (10 µM; Invitrogen; Table 1), 6.8 µl RNase-free water and 2 µl cDNA template. The PCR reactions were performed on a C1000™ Thermal Cycler, CFX96™ Real-Time PCR Detection System (Bio-Rad Laboratories, Inc.) using an activation step at 95 °C for 5 min, and 60 cycles of 95 °C for 20 s and 60 °C for 40 s. As a positive control, a synthetic plasmid containing the target sequence of the TaqMan real-time PCR assay was used. The plasmid contained the target sequence, spanning the nucleotides 1102–1231 of the *Rickettsia rickettsii gltA* gene (GenBank: U59729), synthesized and cloned into pUC57 vector (GenScript).

To determine *Rickettsia* species of the samples positive in the TaqMan real-time PCR assay, conventional PCR assays were used to amplify a fragment of the genes coding for the outer membrane protein B, *ompB*, 17-kDa gene or using semi-nested PCR targeting the *gltA* gene (Table 1), as previously described [35–37].

### Detection of *Babesia* species

Detection of *Babesia* spp. was done using a SYBR green real-time PCR assay, as previously described [38]. Primers BJ1 and BN2 were designed to target the *Babesia 18S* rRNA gene to amplify a 411–452 bp long amplicon depending on the species of *Babesia* [39] (Table 1).

A 20-µl reaction consisted of 10 µl SYBR™ Green PCR Master Mix (Thermo Fisher Scientific), 0.4 µl of each primer (10 µM; Invitrogen; Table 1), 7.2 µl RNase-free water and 2 µl cDNA template. The PCR reactions were performed on a C1000™ Thermal Cycler, CFX96™ Real-Time PCR Detection System (Bio-Rad Laboratories Inc.) using an activation step at 94 °C for 10 min, and 35 cycles of 94 °C for 1 min, 55 °C for 1 min and 72 °C for 2 min, and finally one cycle of 72 °C for 5 min. Immediately after completion of PCR, melting curve analyses were performed by heating to 95 °C for 15 s, followed by cooling to 60 °C for 1 min, and subsequent heating to 95 °C at 0.8 °C min^−1^ with continuous fluorescence recording. As a positive control, a synthetic plasmid containing the target sequence of the SYBR green real-time PCR assay was used. The plasmid contained the target sequence, spanning the nucleotides 467–955 of the *B. divergens 18S* rRNA gene (GenBank: AJ439713), synthesized and cloned into pUC57 vector (GensSript).

### Nucleotide sequencing of PCR-products

Nucleotide sequencing of the PCR products amplified by conventional PCR assays to determine species of *Borrelia* and *Rickettsia* was performed by Macrogen Inc. (Amsterdam, The Netherlands). All sequences were confirmed by sequencing both strands. The obtained chromatograms were initially edited and analyzed using BioEdit Software v7.0 (Tom Hall, Ibis Therapeutics, Carlsbad, CA), and the sequences were examined using Basic Local Alignment Tool (BLAST). The appearance of dual peaks in chromatograms was regarded as a mixed infection of at least two strains of the same species and thus denoted as ‛mixedʼ. An additional file shows all the aligned sequences (see Additional file 1).

### Statistical analyses

Data were presented as percentage for categorical variables, i.e. prevalence of *Borrelia*-positive ticks. The categorical variables were analysed using Chi-square test, but when the expected frequency was < 5 in at least one of the cells of the contingency table we used Fisher’s exact test. The Mann–Whitney test was used to compare cycle threshold (Cq)-values obtained by the real-time PCR assay for the *Borrelia*-positive samples that could be determined to species with the *Borrelia*-positive samples that were denoted as untypeable. Results were reported as median values with interquartile range (IQR). Statistical analyses were performed using GraphPad Prism version 8.0.0 for Windows (GraphPad Software, San Diego, CA). *P*-values ≤ 0.05 were considered statistically significant.

## Results

### Tick collection

In July–August 2015 and May–July 2016, a total of 277 tick specimens was collected by the cloth-dragging method at seven mixed woodland, island localities in the Bothnian Bay area, province of Norrbotten, northern Sweden (Table 2): Axelsvik (*n* = 3); Båtskärsnäs (*n* = 2); Seskarö (*n* = 1); Stora Hamnskär (*n* = 1); Västra Knivskär (*n* = 42); Östra Knivskär (*n* = 2); and Ytterstlandet (*n* = 226). Of the 277 tick specimens, 276 ticks were morphologically and molecularly identified as *I. persulcatus* and one tick (a nymph) was identified as *I. ricinus*. Among all ticks (including the *I. ricinus* nymph), 5.4% were nymphs (*n* = 15), and 94.6% were adult ticks (*n* = 262; 136 adult males, 126 adult females). A significantly higher proportion of nymphs was collected at Västra Knivskär (19%, 8/42) compared to Ytterstlandet (2%, 4/226, *P* < 0.0001). No other significant geographical differences between the proportions of developmental stages were detected.

**Table 2:** Prevalence of *Borrelia* species in *Ixodes persulcatus* ticks (and one *Ixodes ricinus* nymph) collected by cloth-dragging method

Location	Stage^a^	No. of ticks examined	No. (%) of positive ticks	No. (%) of positive ticks containing *Borrelia* species determined by nucleotide sequencing				
				*B. afzelii*	*B. garinii*	*B. valaisiana*	Mixed	Untypeable
Axelsvik	AM	1	0					
	AF	1	0					
	N	1	0					
Båtskärsnäs	AM	2	0					
Seskarö	AF	1	0					
Stora Hamnskär	AM	1	0					
Västra Knivskär	AM	18	3 (17)		1 (33)		1 (33)	1 (33)
	AF	16^c^	2 (13)	1 (50)	1 (50)			
	N	8^b^	3 (43)	1 (33)				2 (66)
Östra Knivskär	N	2^c^	0					
Ytterstlandet	AM	114	71 (62)	30 (42)	29 (40)	1 (2)	1 (2)	10 (14)
	AF	108	71 (66)	37 (52)	21 (30)			13 (18)
	N	4	2 (50)	1 (50)	1 (50)			
All locations	AM	136	74 (54)	30 (41)	30 (41)	1 (1)	2 (2)	11 (15)
	AF	126	73 (58)	38 (52)	22 (30)			13 (18)
	N	15	5 (33)	2 (40)	1 (20)			2 (40)
Total no. of specimens		277	152 (55)	70 (46)	53 (35)	1 (1)	2 (1)	26 (17)

^a^Tick developmental stage: AM, adult male; AF, adult female; N, nymph

^b^One nymph in this group was morphologically and molecularly identified as *Ixodes ricinus.* This nymph was negative for *Borrelia*

^c^One adult female from Västra Knivskär was positive for *Rickettsia helvetica* and one nymph from Östra Knivskär was positive for *Rickettsia* sp. Both *Rickettsia*-positive ticks were negative for *Borrelia*

From the dog at Ytterstlandet, 8 adult male ticks and 8 adult female ticks were collected. All were morphologically and molecularly identified as *I. persulcatus*. We noted that all adult female ticks contained blood, most likely ingested from the dog. One of the adult male ticks also contained blood in the gut. From the dog at Stora Hamnskär, two adult female ticks were collected, and both were morphologically and molecularly identified as *I. persulcatus*. We noted that these two adult females contained blood, most likely ingested from the dog.

### Prevalence of *Borrelia* bacteria in the ticks

Of the ticks collected by the cloth-dragging method, 55% (152/277) were positive for *Borrelia* (Table 2). There was no significant difference between the proportions of *Borrelia*-infected nymphs (33%, 5/15) and *Borrelia*-infected adult ticks (56%, 147/262), and no significant difference between the proportions of *Borrelia*-infected adult males (54%, 74/136) and *Borrelia*-infected adult females (58%, 73/126).

*Borrelia*-infected ticks were detected only at Västra Knivskär and at Ytterstlandet. A significantly higher proportion of *Borrelia*-infected adult ticks was detected at Ytterstlandet (64%, 142/222) compared to Västra Knivskär (15%, 5/34, *P* < 0.0001). There was no significant difference between the proportion of *Borrelia*-infected nymphs at Ytterstlandet (50%, 2/4) compared to that at Västra Knivskär (38%, 3/8).

Of the adult ticks removed from the dog at Ytterstlandet, 31% (5/16) were positive for *Borrelia*: 38% (3/8) of the adult males; and 25% (2/8) of the adult females. Of the two adult female ticks detached from the dog at Stora Hamnskär, both were negative for borreliae.

### Prevalence of *Borrelia* species in the ticks

Of the ticks collected by the cloth-dragging method, three different *Borrelia* species were identified by sequence analysis of the 5S–23S IGS (Table 2). *B. afzelii* was the predominant species and detected in 46% (70/152) of all *Borrelia*-infected ticks followed by *B. garinii* (35%, 53/152), *B. valaisiana* (1%, 1/152), and mixed infections of different *Borrelia* species (1%, 2/152). Seventeen percent (26/152) of all *Borrelia*-infections were untypeable. Of the samples that were determined to *Borrelia* species, no significant differences in prevalence of *Borrelia* species between nymphs and adult ticks or between adult male ticks and adult female ticks were detected. Furthermore, no significant geographical differences in prevalence of *Borrelia* species were detected.

Of the five *Borrelia*-positive adult ticks removed from the dog at Ytterstlandet, one adult female and one adult male contained *B. afzelii* while the other three *Borrelia*-positive samples were untypeable.

The *Borrelia*-positive samples that were defined as untypeable had a significantly higher Cq-value in the real-time PCR analyses (*n* = 29, median 34.3, IQR 30.4–36.0) compared to *Borrelia*-positive samples that could be determined to species (*n* = 128, median 25.9, IQR 23.6–28.9, *P* < 0.0001).

### Prevalence of *Rickettsia* bacteria in the ticks

Of the ticks collected by cloth-dragging, 0.7% (2/277; one adult female tick and one nymphal tick) were positive for *Rickettsia* (Table 2). The *Rickettsia*-positive adult female tick was collected in July 2016 at Västra Knivskär. Our analysis of the amplified *ompB* and 17-kDa sequences revealed a 100% signature match of *Rickettsia helvetica*, as compared with *R. helvetica* sequences deposited in GenBank (AF123725 and LC379447, respectively). The *Rickettsia*-positive nymph was collected in August 2015 at Östra Knivskär. The amplified *ompB* sequences revealed a 95% signature match of *Rickettsia* sp., as compared with *Rickettsia* sp. sequences detected from the host *Adalia bipunctata* ῾the two-spotted ladybug beetleʼ (AJ582613-AJ582615 and AJ628731) deposited in GenBank. Our attempts to amplify fragments of the genes coding for 17-kDa and *gltA* of this *Rickettsia*-positive sample were unsuccessful.

None of the *Rickettsia*-positive *I. persulcatus* ticks were positive for any other analysed tick-borne microorganisms. The ticks collected from the two dogs (*n* = 18) were all negative for *Rickettsia* spp.

### Prevalence of other tick-borne microorganisms in the ticks

Of the 295 ticks analysed (including the 18 ticks collected from the dogs), all were negative for TBEV, *Borrelia miyamotoi*, *A. phagocytophilum*, *N. mikurensis* and *Babesia* spp.

## Discussion

### *Borrelia* infection prevalence

This is the first time that specimens of *I. persulcatus* from Sweden have been analysed for the presence of *Borrelia* species and other potentially human-pathogenic microorganisms. In comparison with previous investigations on the *Borrelia* infection prevalence (36%) of adult female *I. ricinus* in southern and central Sweden [40] the present, significantly higher prevalence (58%) in adult female *I. persulcatus* is noteworthy. Even more so is the difference in *Borrelia* infection prevalence between *I. ricinus* nymphs (25%; [40]) compared to that of adult females of *I. persulcatus* (58%) in this study. The reason for comparing the infection prevalence of *I. ricinus* nymphs with that of *I. persulcatus* adult females is, as explained below, because the nymphal stage of *I. ricinus* and the female adult stage of *I. persulcatus* are the main vectors of human pathogens to humans in northern Europe [1, 3, 4, 13].

Males of *I. persulcatus* may attach to humans and imbibe blood [4]. Our observation of one blood-fed male of *I. persulcatus* collected from a dog at Ytterstlandet, confirms this statement of Pomerantzev (1950) [4].

In a study on *I. ricinus* ticks that had bitten humans in southern and central Sweden and on the Åland archipelago, Finland, Wilhelmsson et al. [40] found that 26% of the ticks were *Borrelia-*infected; they recorded seven different *Borrelia* species; *B. afzelii* was the predominant species and found in 50% of all *Borrelia-*infected ticks, followed by *B. garinii* (19%) and *B. valaisiana* (7%); *B. afzelii* was more prevalent in nymphs (67%) than in adult ticks (46%) whereas *B. garinii* and *B. valaisiana* were more prevalent in adult ticks (30% and 11%, respectively) than in nymphs (20% and 6%, respectively).

In a more recent study on *I. ricinus* and *I. persulcatus* in Finland, Laaksonen et al. [14] found *B. burgdorferi* (*s.l*.) in 18.1% of 1451 *I. persulcatus* ticks analysed and in 16.2% of 2014 *I. ricinus* ticks analysed. In *I. persulcatus*, *B. garinii* (62.6%) was the predominant genospecies followed by *B. afzelii* (35%) and *B. valaisiana* (2.5%). *Borrelia burgdorferi* (*s.s.*) was not found in our study nor in that of Laaksonen et al. [14]. This supports the view that *B. burgdorferi* (*s.s.*) is a relatively rare species in *I. persulcatus* [13, 21]. The prevalence of *B. garinii* (63%) in *I. persulcatus* [14] was even higher than the corresponding prevalence (35%) in our study. However, a relatively large proportion of our *Borrelia*-positive specimens were not possible to identify to genospecies; if they had been possible to identify, it is likely that some of them might have been *B. garinii*.

Of the *Borrelia*-positive ticks, 17% were infected with *Borrelia* that could not be determined to species. These ticks were, in general, infected with a lower amount of *Borrelia* bacteria as indicated by a significantly higher Cq-value, compared to ticks infected by typeable *Borrelia* species. This may, at least partly, explain why PCR products, used to determine *Borrelia* species, were not amplified in the conventional PCR assays.

The lower diversity of potentially human-pathogenic microorganisms recorded in the present populations of *I. persulcatus* in the Baltic Bay area is likely due to several factors: the small total tick sampling area covered; the isolated nature of the islands in the Baltic Bay; the generally low biodiversity, which is a characteristic feature of the Boreal region compared to more southern regions of Sweden and Finland; and the relatively small sample size investigated. The prevalence of TBEV in ticks is usually very low. However, a possible explanation for the negative results obtained in the present study may be the fact that we preserved our ticks in ethanol, which may have permitted degradation of RNA before extracting total NA.

The present study and that of Laaksonen et al. [14] reveal high prevalence of *B. garinii* in *I. persulcatus* in northern Sweden and northern Finland. Neuroborreliosis is usually due to a *B. garinii* infection and is, in general, the most serious of the disease syndromes caused by members of the *B. burgdorferi* (*s.l*.) complex. Consequently, the high prevalence of *B. garinii* in the taiga tick populations in northern Sweden and Finland may constitute a potentially serious public health threat in areas where the taiga tick is abundant.

### Vector behaviour

It is well known that it is usually the nymphs of *I. ricinus* but the adult females of *I. persulcatus*, which attack humans and transmit pathogens to humans [1, 3, 4, 13]. It is also well known that the prevalence of borreliae and the TBEV, in general, is significantly lower in *I. ricinus* than in *I. persulcatus* [13, 14]. There is presumably more than one explanation for these differences. One may be that the questing behaviour of the immature ticks might differ between *I. ricinus* and *I. persulcatus*. For instance, in the Nearctic different nymphal questing behaviours of *I. scapularis*, especially regarding the questing height of nymphs, between northern and southern allopatric populations in the eastern USA, seem to explain the higher Lyme disease risk in areas inhabited by the northern tick population, having nymphs which tend to quest above the leaf litter [41]. Similar differences might exist between the immatures of *I. persulcatus* and *I. ricinus.*

An additional explanation may be that the “host preferences”, i.e. the force of attraction to particular host species may differ between the two Palaearctic tick species. Small and medium-sized mammals constitute the main vertebrate reservoir for *B. afzelii* whereas birds constitute the main vertebrate reservoir for *B. garinii* [42, 43]. Since *I. persulcatus* immature ticks seem to “avoid” or rarely feed on large mammals, which in general are incompetent reservoirs for *B. burgdorferi* (*s.l*.), it may be concluded that, most likely, the pre-adult ticks of *I. persulcatus* feed on small and medium-sized mammals and ground-inhabiting birds, which in general are reservoir-competent for *B. burgdorferi* (*s.l*.). This may be the most likely explanation for the generally higher *Borrelia* infection rate in *I. persulcatus* than in *I. ricinus*.

In *I. ricinus*, on the other hand, larvae, nymphs and adult males and females can usually be found together on large and medium-sized mammals. Therefore, since the immature ticks of *I. ricinus* to a much greater extent, compared to the immatures of *I. persulcatus*, feed on both reservoir-competent and reservoir-incompetent hosts the force of infection of *B. burgdorferi* (*s.l*.) will be weaker, i.e. more “diluted” in *I. ricinus* than in *I. persulcatus*.

In *I. persulcatus*, the adult female tick is the most important vector of pathogens to humans. The adult females of *I. persulcatus* have been described as exhibiting an “aggressive” behaviour, i.e. showing strong attraction towards humans and other large mammals and as being significantly more active than females of *I. ricinus*; this behaviour likely renders them more competent vectors of human pathogens [44].

In contrast to the immature stages of *I. ricinus*, the larvae and nymphs of *I. persulcatus* are rarely found on humans [13] but are usually infesting small mammals and ground-frequenting birds; the nymphs can also be found on medium-sized mammals [13]. Therefore, these “host preferences” of *I. persulcatus* differ from those of its close relative *I. ricinus.*

Since both nymphs and adult females of *I. ricinus* are strongly attracted to feed on humans (and other large mammals) it is nymphs of *I. ricinus*, due to their relatively higher abundance compared to that of adult female ticks, which are the main vectors of pathogens to humans. In *I. persulcatus*, since the nymphs rarely feed on humans it is the adult females, which are strongly attracted to humans, which are the main vectors of pathogens to humans.

*Borrelia afzelii* was the most prevalent (46%) of the *Borrelia*-infected ticks. Small and medium-sized mammals constitute the main vertebrate reservoir for *B. afzelii* [42, 43] and the larvae and nymphs of *I. persulcatus* feed, in general, on small and medium-sized mammals and ground-inhabiting birds. The fauna of small and medium-sized mammals at Ytterstlandet and the other islands, where tick sampling took place, has not been studied in detail but these abundant small mammal species, presumably occur there: *Myodes glareolus*, *M. rufocanus*, *Microtus agrestis* and *Sorex araneus* [45]. One or more of these small mammal species are proven competent reservoirs and transmission hosts for *B. afzelii* [42, 46] and other pathogens vectored by *I. persulcatus*. It is concluded that the feeding behaviour of immatures of *I. persulcatus*, i.e. that small mammals constitute a “preferred” blood source, and that they, in general, are competent reservoirs and transmission hosts for *B. afzelii*, explain the high prevalence of *B. afzelii* in *I. persulcatus* in the study area.

### Geese are presumably a main tick host and a main vertebrate *Borrelia* reservoir

Dense populations of Greylag goose (*Anser anser*) and Canada goose (*Branta canadensis*) breed in the same area of Ytterstlandet where most of the *I. persulcatus* ticks were found. The seasonal taiga tick activity peak, i.e. about April–June, coincides with the breeding time of the geese. Moreover, the first author was told by one of the inhabitants of the island that “a goose that was shot in May 2015 was teeming with ticks”. People who have summer cottages at Ytterstlandet regard the relatively recent massive infestation of the island by the taiga tick to be due to the abundance of geese, which use certain parts of the island for breeding and feeding during April, May and June. These particular parts of the island are the ones most heavily infested by the taiga tick (T.G.T. Jaenson, unpublished observation). *Borrelia garinii* is known to have different birds as its vertebrate reservoir [42, 43, 46]. Together, these observations suggest that geese are a main vertebrate reservoir for *B. garinii* and a main blood host of *I. persulcatus* at Ytterstlandet and some neighbouring islands.

## Conclusions

To our knowledge, this is the first time that *I. persulcatus* from Sweden has been analysed for the presence of tick-borne pathogens. The examined tick populations had a low diversity of tick-borne pathogens but a high prevalence of *B. afzelii* and *B. garinii*.

## Supplementary information

**Additional file 1.** Aligned *Borrelia* and *Rickettsia* nucleotide sequences based on PCR-products.

## Data Availability

The data supporting the conclusions of this article are included within the article. Raw data can be shared with researchers upon a specific request. An additional file shows the aligned *Borrelia* and *Rickettsia* nucleotide sequences (see Additional file 1).
