# Supplementary material for: First records of tick-borne pathogens in populations of the taiga tick Ixodes persulcatus in Sweden
Source: Parasit Vectors. 2019 Nov 28;12:559. doi: 10.1186/s13071-019-3813-0 (PMC6882324; doi:10.1186/s13071-019-3813-0)
Supplement: Supplementary file 1 — Additional file 1. Aligned Borrelia and Rickettsia nucleotide sequences based on PCR-products. [file 13071_2019_3813_MOESM1_ESM.docx]

**Additional file 1. Aligned *Borrelia* and *Rickettsia* nucleotide sequences based on PCR-products**

The identifier line, which begins with '>', gives the name of the **sample ID_microorganism_gene target_tick species_developmental stage of tick_collection method_location of collection**.

**>A1PW471a_Borrelia afzelii_5S-23S ribosomal RNA intergenic spacer, partial sequence_Ixodes persulcatus_adult male_cloth-dragging_ytterstlandet**

GAGTTCGCGGGAGAGTAAGTTATTGCCAGGGTTTTTATTTTATACTTTAAACCTTGAATTTATTTTTTAAATGTTTATATTATTTGAATAAAACATTCAAATAATATAAAAAATAAAATATATATTGACATGGATTAAACAAAGATATATATTATTTTATGTTGTATGAACAAATTGGCAAAATAGAGATGGAAGATAAAAATATGGTCAAAGTAATAAGAGTCTATGG

**>A2PW471b_Borrelia afzelii_5S-23S ribosomal RNA intergenic spacer, partial sequence_Ixodes persulcatus_adult male_cloth-dragging_ytterstlandet**

GAGTTCGCGGGAGAGTAAGTTATTGCCAGGGTTTTTATTTTATACTTTAAACCTTGAATTTATTTTTTAAATGTTTATATTATTTGAATAAAACATTCAAATAATATAAAAAATAATATATATATTGACATGGATTAAACAAAGATATATATTATTTTATGTTGTATAAACAAATTGGCAAAATAGAGATGGAAGATAAAAATATGGTCAAAGTAATAAGAGTCTATGGTGAATGCCTA

**>A3PW470a_Borrelia afzelii_5S-23S ribosomal RNA intergenic spacer, partial sequence_Ixodes persulcatus_adult female_cloth-dragging_ytterstlandet**

GAGTTCGCGGGAGAGTAAGTTATTGCCAGGGTTTTTATTTTATACTTTAAACCTTGAATTTATTTTTTAAATGTTTATATTATTTGAATAAAACATTCAAATAATATAAAAAATAATATATATATTGACATGGATTAAACAAAGATATATATTATTTTATGTTGTATAAACAAATTGGCAAAATAGAGATGGAAGATAAAAATATGGTCAAAGTAATAAGAGTCTATGGTGAATGCC

**>A4PW470b_Borrelia garinii_5S-23S ribosomal RNA intergenic spacer, partial sequence_Ixodes persulcatus_adult female_cloth-dragging_ytterstlandet**

GGTTTTTATTTTATACTTTAAACATTGATTTTATTTTTTATGTTTTTAGATGTTCATGTTTTTGAATGTTTTATTTAAATAATATAAAAAATAAAATATATATTGACATGGATTAAACAAAGATATATATTATTCTATGTTGCATAAGCAAATTGGCAAAATAGAGATGGAAGATAAAAATATGGTCAAAGTAATAAGAGTCTATGGTGAATGCCTAGGA

**>A5PW470c_Borrelia afzelii_5S-23S ribosomal RNA intergenic spacer, partial sequence_Ixodes persulcatus_adult female_cloth-dragging_ytterstlandet**

GAGTTCGCGGGAGAGTAAGTTATTGCCAGGGTTTTTATTTTATACTTTAAACCTTGAATTTATTTTTTAAATGTTTATATTATTTGAATAAAACATTCAAATAATATAAAAAATAATATATATATTGACATGGATTAAACAAAGATATATATTATTCTATGTTGTATGAACAAATTGGCAAAATAGAGATGGAAGATAAAAATATGGTCAAAGTAATAAGAGTCTATGGTGAATGCC

**>A6PW472c_Borrelia garinii_5S-23S ribosomal RNA intergenic spacer, partial sequence_Ixodes persulcatus_adult female_cloth-dragging_ytterstlandet**

GAGTTCGCGGGAGAGTAAGTTATTGCCAGGGTTTTTATTTTATACTTTAAACATTGATCTTATTTTTTATGTTTTTAGATGTTCATGTTTTTGAATGTTTTATTTAAATAATATAAAAAATAAAATATATATTGACATGGATTAAACAAAGATATATATTATTCTATGTTGCATAAACAAATTGGCAAAATAGAGATGGAAGATAAAAATATGGTCAAAGTAATAAGAGTCTATGGTGAATGCC

**>A7PW472d_Borrelia garinii_5S-23S ribosomal RNA intergenic spacer, partial sequence_Ixodes persulcatus_adult female_cloth-dragging_ytterstlandet**

CTGCGAGTTCGCGGGAGAGTAAGTTATTGCCAGGGTTTTTATTTTATACTTTAAACATTGATTTTATTTTTTATGTTTTTAGATTTTCATGTTTTTAGATTTTCATGTTTTTTAATGTTTTATTTAAATAATATAAAAAATAAAATATATATATTGACATGGATTAAACAAAGATATATATTATTCTATGTTGCATAAACAAATTGGCAAAATAGAGATGGAAGATAAAAATATGGTCAAAGTAATAAGAGTCTATGGTGAATGCCTAGGA

**>A8PW473b_Borrelia garinii_5S-23S ribosomal RNA intergenic spacer, partial sequence_Ixodes persulcatus_adult male_cloth-dragging_ytterstlandet**

GAGTAAGTTATTGCCAGGGTTTTTATTTTATACTTTAAACATTGATTTTATTTTTTATGTTTTTAGATGTTCATGTTTTTGAATGTTTTATTTAAATAATATAAAAAATAAAATATATATTGACATGGATTAAACAAAGATATATATTATTCTATGTTGCATAAACAAATTGGCAAAATAGAGATGGAAGATAAAAATATGGTCAAAGTAATAAGAGTCTA

**>A9PW473c_UNTYPEABLE_5S-23S ribosomal RNA intergenic spacer, partial sequence_Ixodes persulcatus_adult male_cloth-dragging_ytterstlandet**

No sequences obtained

**>A10PW473d_UNTYPEABLE_5S-23S ribosomal RNA intergenic spacer, partial sequence_Ixodes persulcatus_adult male_cloth-dragging_ytterstlandet**

No sequences obtained

**>A11PW473e_Borrelia garinii_5S-23S ribosomal RNA intergenic spacer, partial sequence_Ixodes persulcatus_adult male_cloth-dragging_ytterstlandet**

TTTTTTATGTTTTTAGATGTTCATGTTTTTGAATGTTTTATTTAAATAATATAAAAAATAAAATATATATTGACATGGATTAAACAAAGATATATATTATTCTATGTTGCATAAACAAATTGGCAAAATAGAGATGGAAGATAAAAATATGGTCAAAGTAATAAGAGTCTATGGTGAATGCCTAAAAAAA

**>A12PW473f_Borrelia garinii_5S-23S ribosomal RNA intergenic spacer, partial sequence_Ixodes persulcatus_adult male_cloth-dragging_ytterstlandet**

GAGTTCGCGGGAGAGTAAGTTATTGCCAGGGTTTTTATTTTATACTTTAAACATTGATTTTATTTTTTATGTTTTTAGATGTTCATGTTTTTGAATGTTTTATTTAAATAATATAAAAAATAAAATATATATTGACATGGATTAAACAAAGATATATATTATTCTATGTTGCATAAACAAATTGGCAAAATAGAGATGGAAGATAAAAATATGGTCAAAGTAATAAGAGTCTATGGT

**>B1PW474a_Borrelia afzelii_5S-23S ribosomal RNA intergenic spacer, partial sequence_Ixodes persulcatus_adult female_cloth-dragging_ytterstlandet**

GAGTTCGCGGGAGAGTAAGTTATTGCCAGGGTTTTTATTTTATACTTTAAACCTTGAATTTATTTTTTAAATGTTTATATTATTTGAATAAAACATTCAAATAATATAAAAAATAATATATATATTGACATGGATTAAACAAAGATATATATTATTTTATGTTGTATAAACAAATTGGCAAAATAGAGATGGAAGATAAAAATATGGTCAAAGTAATAAGAGTCTATGGTGAATGCCTA

**>B2PW474b_UNTYPEABLE_5S-23S ribosomal RNA intergenic spacer, partial sequence_Ixodes persulcatus_adult female_cloth-dragging_ytterstlandet**

No sequences obtained

**>B3PW474d_Borrelia garinii_5S-23S ribosomal RNA intergenic spacer, partial sequence_Ixodes persulcatus_adult female_cloth-dragging_ytterstlandet**

GGGAGAGTAAGTTATTGCCAGGGTTTTTATTTTATACTTTAAACATTGATTTTATTTTTTATGTTTTTAGATATTTATGTTTTTGAATGTTTTATTCAAATAATATAAAAAATAAAATATATATTGACATGGATTAAACAAAGATATATATTATTCTATGTTGTATAAACAAATTGGCAAAATAGAGATGGAAGATAAAAATATGGTCAAAGTAATAAGAGTCTATGGTGAATGCCTA

**>B4PW484a_Borrelia afzelii_5S-23S ribosomal RNA intergenic spacer, partial sequence_Ixodes persulcatus_adult female_cloth-dragging_ytterstlandet**

GAGTTCGCGGGAGAGTAAGTTATTGCCAGGGTTTTTATTTTATACTTTAAACCTTGAATTTATTTTTTAAATGTTTATATTATTTGAATAAAACATTCAAATAATATAAAAAATAATATATATATTGACATGGATTAAACAAAGATATATATTATTCTATGTTGTATGAACAAATTGGCAAAATAGAGATGGAAGATAAAAATATGGTCAAAGTAATAAGAGTCTATGGTGAATGCCTA

**>B5PW484b_Borrelia afzelii_5S-23S ribosomal RNA intergenic spacer, partial sequence_Ixodes persulcatus_adult female_cloth-dragging_ytterstlandet**

GAGTTCGCGGGAGAGTAAGTTATTGCCAGGGTTTTTATTTTATACTTTAAACCTTGAATTTATTTTTTAAATGTTTATATTATTTGAATAAAACATTCAAATAATATAAAAAATAATATATATATTGACATGGATTAAACAAAGATATATATTATTCTATGTTGTATGAACAAATTGGCAAAATAGAGATGGAAGATAAAAATATGGTCAAAGTAATAAGAGTCTATGGTGAATGCCTA

**>B6PW475c_Borrelia afzelii_5S-23S ribosomal RNA intergenic spacer, partial sequence_Ixodes persulcatus_adult male_cloth-dragging_ytterstlandet**

GGTTTTTATTTTATACTTTAAACCTTGAATTTATTTTTTAAATGTTTATATTATTTGAATAAAACATTCAAATAATATAAAAAATAATATATATATTGACATGGATTAAACAAAGATATATATTATTTTATGTTGTATAAACAAATTGGCAAAATAGAGATGGAAGATAAAAATATGGTCAAAGTAATAAGAGTCTATGGTGAATGCCTAGGAGCTT

**>B7PW475d_Borrelia afzelii_5S-23S ribosomal RNA intergenic spacer, partial sequence_Ixodes persulcatus_adult male_cloth-dragging_ytterstlandet**

GGTTTTTTTTTTTTACTTTAACCTTGAATTTATTTTTTAAATGTTTATATTATTTGAATAAAACATTCAAATAATATAAAAAATAATATATATATTGACATGGATTAAACAAAGATATATATTATTCTATGTTGTATGAACAAATTGGCAAAATAGAGATGGAAGATAAAAATATGGTCAAAGTAATAAGAGTCTATGGTGAATGCCTA

**>B8PW476a_Borrelia garinii_5S-23S ribosomal RNA intergenic spacer, partial sequence_Ixodes persulcatus_adult male_cloth-dragging_ytterstlandet**

TTATACTTTAAACATTGATTTTATTTTTTATGTTTTTAGATGTTCATGTTTTTGAATGTTTTATTTAAATAATATAAAAAATAAAATATATATTGACATGGATTAAACAAAGATATATATTATTCTATGTTGCATAAACAAATTGGCAAAATAGAGATGGAAGATAAAAATATGGTCAAAGTAATAAGAGTCTATGGTGAATGCCTA

**>B9PW476b_Borrelia garinii_5S-23S ribosomal RNA intergenic spacer, partial sequence_Ixodes persulcatus_adult male_cloth-dragging_ytterstlandet**

TTTATTTTATACTTTAAACATTGATTTTATTTTTTATGTTTTTAGATGTTCATGTTTTTGAATGTTTTATTTAAATAATATAAAAAATAAAATATATATTGACATGGATTAAACAAAGATATATATTATTCTATGTTGCATAAACAAATTGGCAAAATAGAGATGGAAGATAAAAATATGGTCAAAGTAATAAGAGTCTATGGTGAATGCCTA

**>B10PW476c_Borrelia garinii_5S-23S ribosomal RNA intergenic spacer, partial sequence_Ixodes persulcatus_adult male_cloth-dragging_ytterstlandet**

CATTGATTTTATTTTTTATGTTTTTAGATGTTCATGTTTTTGAATGTTTTATTTAAATAATATAAAAAATAAAATATATATTGACATGGATTAAACAAAGATATATATTATTCTATGTTGCATAAGCAAATTGGCAAAATAGAGATGGAAGATAAAAATATGGTCAAAGTAATAAGAGTCTATGGTGAATGCCTA

**>B11PW476d_Borrelia garinii_5S-23S ribosomal RNA intergenic spacer, partial sequence_Ixodes persulcatus_adult male_cloth-dragging_ytterstlandet**

ACATTGATTTTATTTTTTATGTTTTTAGATGTTCATGTTTTTGAATGTTTTATTTAAATAATATAAAAAATAAAATATATATTGACATGGATTAAACAAAGATATATATTATTCTATGTTGCATAAACAAATTGGCAAAATAGAGATGGAAGATAAAAATATGGTCAAAGTAATAAGAGTCTATGGTGAATGCCTA

**>B12PW477a_Borrelia afzelii_5S-23S ribosomal RNA intergenic spacer, partial sequence_Ixodes persulcatus_adult female_cloth-dragging_ytterstlandet**

ACCTTGAATTTATTTTTTAAATGTTTATATTATTTGAATAAAACATTCAAATAATATAAAAAATAATATATATATTGACATGGATTAAACAAAGATATATATTATTCTATGTTGTATGAACAAATTGGCAAAATAGAGATGGAAGATAAAAATATGGTCAAAGTAATAAGAGTCTATGGTGAATGCCTA

**>C1PW477b_Borrelia afzelii_5S-23S ribosomal RNA intergenic spacer, partial sequence_Ixodes persulcatus_adult female_cloth-dragging_ytterstlandet**

CTTTAACCTTGAATTTATTTTTTAAATGTTTATATTATTTGAATAAAACATTCAAATAATATAAAAAATAATATATATATTGACATGGATTAAACAAAGATATATATTATTTTATGTTGTATAAACAAATTGGCAAAATAGAGATGGAAGATAAAAATATGGTCAAAGTAATAAGAGTCTATGGTGAATGCCTA

**>C2PW477f_Borrelia afzelii_5S-23S ribosomal RNA intergenic spacer, partial sequence_Ixodes persulcatus_adult female_cloth-dragging_ytterstlandet**

TTTATTTTTTAAATGTTTATATTATTTGAATAAAACATTCAAATAATATAAAAAATAATATATATATTGACATGGATTAAACAAAGATATATATTATTTTATGTTGTATAAACAAATTGGCAAAATAGAGATGGAAGATAAAAATATGGTCAAAGTAATAAGAGTCTATGGTGAATGCCTA

**>C3PW477g_UNTYPEABLE_5S-23S ribosomal RNA intergenic spacer, partial sequence_Ixodes persulcatus_adult female_cloth-dragging_ytterstlandet**

No sequences obtained

**>C4PW485_Borrelia afzelii_5S-23S ribosomal RNA intergenic spacer, partial sequence_Ixodes persulcatus_nymph_cloth-dragging_ytterstlandet**

CCTTGAATTTATTTTTTAAATGTTTATATTATTTGAATAAAACATTCAAATAATATAAAAAATAATATATATATTGACATGGATTAAACAAAGATATATATTATTTTATGTTGTATAAACAAATTGGCAAAATAGAGATGGAAGATAAAAATATGGTCAAAGTAATAAGAGTCTATGGTGAATGCCTAGGAGC

**>C5PW480c_Borrelia afzelii_5S-23S ribosomal RNA intergenic spacer, partial sequence_Ixodes persulcatus_adult female_cloth-dragging_västra knivskär**

ATTTATTTTTTAATGTTTATATTATTTGAATGTTTTATTCAAATAATATAAAAAATAATATATATATTGACATGGATTAAACAAAGATATATATTATTCTATGTTGTATAAACAAATTGGCAAAATAGAGATGGAAGATAAAAATATGGTCAAAGTAATAAGAGTCTATGGTGAATGCC

**>C6PW481a_Borrelia garinii_5S-23S ribosomal RNA intergenic spacer, partial sequence_Ixodes persulcatus_adult male_cloth-dragging_västra knivskär**

ATACTTCAAACATTGATTTTATTTTTTATGTTTTTAGATGTTCATGTTTTTGAATGTTTTATTCGAATAATATAAAAAATAAAATATATATTGACATGGATTAAACAAAGATATATATTATTCTATGTTGTATAAACAAATTGGCAAAATAGAGATGGAAGATAAAAATATGGTCAAAGTAATAAGAGTCTATGGTGAATGCC

**>C7PW481c_UNTYPEABLE_5S-23S ribosomal RNA intergenic spacer, partial sequence_Ixodes persulcatus_adult male_cloth-dragging_västra knivskär**

No sequences obtained

**>C8PW481d_mixed_5S-23S ribosomal RNA intergenic spacer, partial sequence_Ixodes persulcatus_adult male_cloth-dragging_västra knivskär**

Dual peaks in the chromatograms

**>C9PW482a_UNTYPEABLE_5S-23S ribosomal RNA intergenic spacer, partial sequence_Ixodes persulcatus_nymph_cloth-dragging_västra knivskär**

No sequences obtained

**>C10PW482b_Borrelia afzelii_5S-23S ribosomal RNA intergenic spacer, partial sequence_Ixodes persulcatus_nymph_cloth-dragging_västra knivskär**

ATTTATTTTTTAAATGTTTATATTATTTGAATGTTTTATTCAAATAATATAAAAAATAATATATATATTGACATGGATTAAACAAAGATATATATTATTCTATGTTGTATAAACAAATTGGCAAAATAGAGATGGAAGATAAAAATATGGTCAAAGTAATAAGAGTCTATGGTGAATGCCTA

**>C11PW482c_UNTYPEABLE_5S-23S ribosomal RNA intergenic spacer, partial sequence_Ixodes persulcatus_nymph_cloth-dragging_västra knivskär**

No sequences obtained

**>C12PW483a_UNTYPEABLE_5S-23S ribosomal RNA intergenic spacer, partial sequence_Ixodes persulcatus_adult male_cloth-dragging_ytterstlandet**

No sequences obtained

**>D1PW483b_Borrelia afzelii_5S-23S ribosomal RNA intergenic spacer, partial sequence_Ixodes persulcatus_adult male_cloth-dragging_ytterstlandet**

CTTTAAACCTTGAATTTATTTTTTAAATGTTTATATTATTTGAATAAAACATTCAAATAATATAAAAAATAATATATATATTGACATGGATTAAACAAAGATATATATTATTCTATGTTGTATGAACAAATTGGCAAAATAGAGATGGAAGATAAAAATATGGTCAAAGTAATAAGAGTCTATGGTGAATGCCTA

**>D2PW483c_Borrelia garinii_5S-23S ribosomal RNA intergenic spacer, partial sequence_Ixodes persulcatus_adult male_cloth-dragging_ytterstlandet**

AACATTGATTTTATTTTTTATGTTTTTAGATGTTCATGTTTTTGAATGTTTTATTTAAATAATATAAAAAATAAAATATATATTGACATGGATTAAACAAAGATATATATTATTCTATGTTGCATAAACAAATTGGCAAAATAGAGATGGAAGATAAAAATATGGTCAAAGTAATAAGAGTCTATGGTGAATGCCTA

**>D3PW487a_Borrelia garinii_5S-23S ribosomal RNA intergenic spacer, partial sequence_Ixodes persulcatus_adult female_cloth-dragging_ytterstlandet**

AACATTGATTTTATTTTTTATGTTTTTAGATGTTCATGTTTTTGAATGTTTTATTTAAATAATATAAAAAATAAAATATATATTGACATGGATTAAACAAAGATATATATTATTCTATGTTGCATAAGCAAATTGGCAAAATAGAGATGGAAGATAAAAATATGGTCAAAGTAATAAGAGTCTATGGTGAATGCCTA

**>D4PW488a_Borrelia garinii_5S-23S ribosomal RNA intergenic spacer, partial sequence_Ixodes persulcatus_adult male_cloth-dragging_ytterstlandet**

TATACTTTAAACATTGATTTTATTTTTTATGTTTTTAGATGTTCATGTTTTTGAATGTTTTATTTAAATAATATAAAAAATAAAATATATATTGACATGGATTAAACAAAGATATATATTATTCTATGTTGCATAAGCAAATTGGCAAAATAGAGATGGAAGATAAAAATATGGTCAAAGTAATAAGAGTCTATGGTGAATGCCTAG

**>D5PW489a_Borrelia afzelii_5S-23S ribosomal RNA intergenic spacer, partial sequence_Ixodes persulcatus_adult female_cloth-dragging_ytterstlandet**

TTATACTTTAAACTTGAATTTATTTTTTAAATGTTTATATTATTTGAATAGATATATCATTCAAATAATATAAAAAATAATATATATATTGACATGGATTAAACAAAGATATATATTATTCTATGTTGTATAAACAAATTGGCAAAATAGAGATGGAAGATAAAAATATGGTCAAAGTAATAAGAGTCTATGGTGAATGC

**>D6PW489c_Borrelia garinii_5S-23S ribosomal RNA intergenic spacer, partial sequence_Ixodes persulcatus_adult female_cloth-dragging_ytterstlandet**

TTTATTTTTTATGTTTTTAGATGTTCATGTTTTTGAATGTTTTATTTAAATAATATAAAAAATAAAATATATATTGACATGGATTAAACAAAGATATATATTATTCTATGTTGCATAAGCAAATTGGCAAAATAGAGATGGAAGATAAAAATATGGTCAAAGTAATAAGAGTCTATGGT

**>D7PW489d_Borrelia garinii_5S-23S ribosomal RNA intergenic spacer, partial sequence_Ixodes persulcatus_adult female_cloth-dragging_ytterstlandet**

TTATACTTTAAACATTGATTTTATTTTTTATGTTTTTAGATGTTCATGTTTTTGAATGTTTTATTTAAATAATATAAAAAATAAAATATATATTGACATGGATTAAACAAAGATATATATTATTCTATGTTGCATAAACAAATTGGCAAAATAGAGATGGAAGATAAAAATATGGTCAAAGTAATAAGAGTCTATGGTGATGCCTA

**>D8PW489e_UNTYPEABLE_5S-23S ribosomal RNA intergenic spacer, partial sequence_Ixodes persulcatus_adult female_cloth-dragging_ytterstlandet**

No sequences obtained

**>D9PW490c_UNTYPEABLE_5S-23S ribosomal RNA intergenic spacer, partial sequence_Ixodes persulcatus_adult female_cloth-dragging_ytterstlandet**

No sequences obtained

**>D10PW490e_Borrelia afzelii_5S-23S ribosomal RNA intergenic spacer, partial sequence_Ixodes persulcatus_adult female_cloth-dragging_ytterstlandet**

CCTTGAATTTATTTTTTAAATGTTTATATTATTTGAATAAAACATTCAAATAATATAAAAAATAATATATATATTGACATGGATTAAACAAAGATATATATTATTCTATGTTGTATGAACAAATTGGCAAAATAGAGATGGAAGATAAAAATATGGTCAAAGTAATAAGAGTCTATGGTGAATGCCTA

**>D11PW490h_Borrelia afzelii_5S-23S ribosomal RNA intergenic spacer, partial sequence_Ixodes persulcatus_adult female_cloth-dragging_ytterstlandet**

CTTTAAACCTTGAATTTATTTTTTAAATGTTTATATTATTTGAATAAAACATTCAAATAATATAAAAAATAATATATATATTGACATGGATTAAACAAAGATATATATTATTCTATGTTGTATGAACAAATTGGCAAAATAGAGATGGAAGATAAAAATATGGTCAAAGTAATAAGAGTCTATGGTGAATGCCTA

**>D12PW490j_UNTYPEABLE_5S-23S ribosomal RNA intergenic spacer, partial sequence_Ixodes persulcatus_adult female_cloth-dragging_ytterstlandet**

No sequences obtained

**>E1PW497c_Borrelia garinii_5S-23S ribosomal RNA intergenic spacer, partial sequence_Ixodes persulcatus_adult male_cloth-dragging_ytterstlandet**

GAGTAAGTTATTGCCAGGGTTTTTATTTTATACTTTAAACATTGATTTTATTTTTTATGTTTTTAGATGTTCATGTTTTTGAATGTTTTATTTAAATAATATAAAAAATAAAATATATATTGACATGGATTAAACAAAGATATATATTATTCTATGTTGCATAAGCAAATTGGCAAAATAGAGATG

**>E2PW491b_Borrelia garinii_5S-23S ribosomal RNA intergenic spacer, partial sequence_Ixodes persulcatus_adult female_cloth-dragging_ytterstlandet**

CATTGATTTTATTTTTTATGTTTTTAGATGTTCATGTTTTTGAATGTTTTATTTAAATAATATAAAAAATAAAATATATATTGACATGGATTAAACAAAGATATATATTATTCTATGTTGCATAAACAAATTGGCAAAATAGAGATGGAAGATAAAAATATGGTCAAAGTAATAAGAGTCTATGGTGAATGCCTA

**>E3PW491c_Borrelia garinii_5S-23S ribosomal RNA intergenic spacer, partial sequence_Ixodes persulcatus_adult female_cloth-dragging_ytterstlandet**

CATTGATTTTATTTTTTATGTTTTTAGATGTTCATGTTTTTGAATGTTTTATTTAAATAATATAAAAAATAAAATATATATTGACATGGATTAAACAAAGATATATATTATTCTATGTTGCATAAACAAATTGGCAAAATAGAGATGGAAGATAAAAATATGGTCAAAGTAATAAGAGTCTATGGTGA

**>E4PW491d_Borrelia garinii_5S-23S ribosomal RNA intergenic spacer, partial sequence_Ixodes persulcatus_adult female_cloth-dragging_ytterstlandet**

AACATTGATTTTATTTTTTATGTTTTTAGATGTTCATGTTTTTGAATGTTTTATTTAAATAATATAAAAAATAAAATATATATTGACATGGATTAAACAAAGATATATATTATTCTATGTTGCATAAACAAATTGGCAAAATAGAGATGGAAGATAAAAATATGGTCAAAGTAATAAGAGTCTATGGTGAATGCCTA

**>E5PW491f_Borrelia afzelii_5S-23S ribosomal RNA intergenic spacer, partial sequence_Ixodes persulcatus_adult female_cloth-dragging_ytterstlandet**

ACTTTAACCTTGAATTTATTTTTTAAATGTTTATATTATTTGAATAAAACATTCAAATAATATAAAAAATAATATATATATTGACATGGATTAAACAAAGATATATATTATTCTATGTTGTATAAACGAATTGGCAAAATAGAGATGGAAGATAAAAATATGGTCAAAGTAATAAGAGTCTATGGTGAATGCCTA

**>E6PW491j_Borrelia afzelii_5S-23S ribosomal RNA intergenic spacer, partial sequence_Ixodes persulcatus_adult female_cloth-dragging_ytterstlandet**

ACTTTAAACCTTAAATTTATTTTTTAAATGTTTATATTATTTGAATAAAACATTCAAATAATATAAAAAATAATATATATATTGACATGGATTAAACAAAGATATATATTATTTTATGTTGTATAAACAAATTGGCAAAATAGAGATGGAAGATAAAAATATGGTCAAAGTAATAAGAGTCTATGGTGAATGCCTA

**>E7PW492a_Borrelia afzelii_5S-23S ribosomal RNA intergenic spacer, partial sequence_Ixodes persulcatus_adult male_cloth-dragging_ytterstlandet**

AACCTTGAATTTATTTTTTAAATGTTTATATTATTTGAATAAAACATTCAAATAATATAAAAAATAATATATATATTGACATGGATTAAACAAAGATATATATTATTCTATGTTGTATGAACAAATTGGCAAAATAGAGATGGAAGATAAAAATATGGTCAAAGTAATAAGAGTCTATGGTGAATGCCTA

**>E8PW492c_Borrelia afzelii_5S-23S ribosomal RNA intergenic spacer, partial sequence_Ixodes persulcatus_adult male_cloth-dragging_ytterstlandet**

TTTATACTTTAAACCTTGAATTTATTTTTTAAATGTTTATATTATTTGAATAAAACATTCAAATAATATAAAAAATAATATATATATTGACATGGATTAAACAAAGATATATATTATTCTATGTTGTATGAACAAATTGGCAAAATAGAGATGGAAGATAAAAATATGGTCAAAGTAATAAGAGTCT

**>E9PW492e_Borrelia garinii_5S-23S ribosomal RNA intergenic spacer, partial sequence_Ixodes persulcatus_adult male_cloth-dragging_ytterstlandet**

TACTTTAAACATTGATTTTATTTTTTATGTTTTTAGATGTTCATGTTTTTGAATGTTTTATTTAAATAATATAAAAAATAAAATATATATTGACATGGATTAAACAAAGATATATATTATTCTATGTTGCATAAGCAAATTGGCAAAATAGAGATGGAAGATAAAAATATGGTCAAAGTAATAAGAGTCT

**>E10PW492f_Borrelia garinii_5S-23S ribosomal RNA intergenic spacer, partial sequence_Ixodes persulcatus_adult male_cloth-dragging_ytterstlandet**

TATACTTTAAACATTGATTTTATTTTTTATGTTTTTAGATGTTCATGTTTTTGAATGTTTTATTTAAATAATATAAAAAATAAAATATATATTGACATGGATTAAACAAAGATATATATTATTCTATGTTGCATAAGCAAATTGGCAAAATAGAGATGGAAGATAAAAATATGGTCAAAGTAATAAGAGTCTATGGTGAATGCCTA

**>E11PW492h_Borrelia afzelii_5S-23S ribosomal RNA intergenic spacer, partial sequence_Ixodes persulcatus_adult male_cloth-dragging_ytterstlandet**

GTTTATATTATTTGAATAAAACATTCAAATAATATAAAAAATAATATATATATTGACATGGATTAAACAAAGATATATATTATTCTATGTTGTATGAACAAATTGGCAAAATAGAGATGGAAGATAAAAATATGGTCAAAGTAATAAGAGTCTATGGTGAATGCCTA

**>E12PW492i_UNTYPEABLE_5S-23S ribosomal RNA intergenic spacer, partial sequence_Ixodes persulcatus_adult male_cloth-dragging_ytterstlandet**

No sequences obtained

**>F1PW376a_Borrelia afzelii_5S-23S ribosomal RNA intergenic spacer, partial sequence_Ixodes persulcatus_adult female_cloth-dragging_ytterstlandet**

AGGGTTTTTATTTTATACTTTAAACCTTGAATTTATTTTTTAAATGTTTATATTATTTGAATAAAACATTCAAATAATATAAAAAATAATATATATATTGACATGGATTAAACAAAGATATATATTATTCTATGTTGTATGAACAAATTGGCAAAATAGAGATGGAAGATAAAAATATGGTCAAAGTAATAAGAGTCTATGGTGAATGCCTA

**>F2PW376b_Borrelia garinii_5S-23S ribosomal RNA intergenic spacer, partial sequence_Ixodes persulcatus_adult female_cloth-dragging_ytterstlandet**

TTATACTTTAAACATTGATTTTATTTTTTATGTTTTTAGATGTTCATGTTTTTGAATGTTTTATTTAAATAATATAAAAAATAAAATATATATTGACATGGATTAAACAAAGATATATATTATTCTATGTTGCATAAACAAATTGGCAAAATAGAGATGGAAGATAAAAATATGGTCAAAGTAATAAGAGTCTATGGTGAATGCCTA

**>F3PW493a_Borrelia garinii_5S-23S ribosomal RNA intergenic spacer, partial sequence_Ixodes persulcatus_adult male_cloth-dragging_ytterstlandet**

GATTTTATTTTTTATATTTTTAGATGTTCATGTTTTTGAATGTTTTATTTAAATAATATAAAAAATAAAATATATATTGACATGGATTAAACAAAGATATATATTATTCTATGTTGCATAAACAAATTGGCAAAATAGAGATGGAAGATAAAAATATGGTCAAAGTAATAAGAGTCT

**>F4PW493c_Borrelia afzelii_5S-23S ribosomal RNA intergenic spacer, partial sequence_Ixodes persulcatus_adult male_cloth-dragging_ytterstlandet**

AACCTTGAATTTATTTTTTAAATGTTTATATTATTTGAATAAAACATTCAAATAATATAAAAAATAATATATATATTGACATGGATTAAACAAAGATATATATTATTCTATGTTGTATGAACGAATTGGCAAAATAGAGATGGAAGATAAAAATATGGTCAAAGTAATAAGAGTCTATGGTGAATGCCTA

**>F5PW493d_mixed_5S-23S ribosomal RNA intergenic spacer, partial sequence_Ixodes persulcatus_adult male_cloth-dragging_ytterstlandet**

Dual peaks in the chromatogram

**>F6PW493e_Borrelia garinii_5S-23S ribosomal RNA intergenic spacer, partial sequence_Ixodes persulcatus_adult male_cloth-dragging_ytterstlandet**

AACATTGATTTTATTTTTTATGTTTTTAGATGTTCATGTTTTTGAATGTTTTATTTAAATAATATAAAAAATAAAATATATATTGACATGGATTAAACAAAGATATATATTATTCTATGTTGCATAAACAAATTGGCAAAATAGAGATGGAAGATAAAAATATGGTCAAAGTAATAAGAGTCTATGGTGAATGCCTA

**>F7PW493f_UNTYPEABLE_5S-23S ribosomal RNA intergenic spacer, partial sequence_Ixodes persulcatus_adult male_cloth-dragging_ytterstlandet**

No sequences obtained

**>F8PW493g_Borrelia garinii_5S-23S ribosomal RNA intergenic spacer, partial sequence_Ixodes persulcatus_adult male_cloth-dragging_ytterstlandet**

AGTTATTGCCAGGGTTTTTATTTTATACTTTAAACATTGATTTTATTTTTTATGTTTTTAGATGTTCATGTTTTTGAATGTTTTATTTAAATAATATAAAAAATAAAATATATATTGACATGGATTAAACAAAGATATATATTATTCTATGTTGCATAAACAAATTGGCAAAATAGAGATGGAAGATAAAAATATGGTCAAAGTAATAAGAGTCTATGGTGAATGCCTA

**>F9PW493h_Borrelia garinii_5S-23S ribosomal RNA intergenic spacer, partial sequence_Ixodes persulcatus_adult male_cloth-dragging_ytterstlandet**

AACATTGATTTTATTTTTTATGTTTTTAGATGTTCATGTTTTTGAATGTTTTATTTAAATAATATAAAAAATAAAATATATATTGACATGGATTAAACAAAGATATATATTATTCTATGTTGCATAAACAAATTGGCAAAATAGAGATGGAAGATAAAAATATGGTCAAAGTAATAAGAGTCTATGGTGAATGCCC

**>F10PW494a_Borrelia afzelii_5S-23S ribosomal RNA intergenic spacer, partial sequence_Ixodes persulcatus_adult female_cloth-dragging_ytterstlandet**

AACCTTGAATTTATTTTTTAAATGTTTATATTATTTGAATAAAACATTCAAATAATATAAAAAATAATATATATATTGACATGGATTAAACAAAGATATATATTATTTTATGTTGTATAAACAAATTGGCAAAATAGAGATGGAAGATAAAAATATGGTCAAAGTAATAAGAGTCTATGGTGAATGCCTA

**>F11PW494b_Borrelia garinii_5S-23S ribosomal RNA intergenic spacer, partial sequence_Ixodes persulcatus_adult female_cloth-dragging_ytterstlandet**

TTATTTTATACTTTAAACATTGATTTTATTTTTTATGTTTTTAGATGTTCATGTTTTTGAATGTTTTATTTAAATAATATAAAAAATAAAATATATATTGACATGGATTAAACAAAGATATATATTATTCTATGTTGCATAAACAAATTGGCAAAATAGAGATGGAAGATAAAAATATGGTCAAAGTAATAAGAGTCTATG

**>F12PW494d_Borrelia garinii_5S-23S ribosomal RNA intergenic spacer, partial sequence_Ixodes persulcatus_adult female_cloth-dragging_ytterstlandet**

AACATTGATTTTATTTTTTATGTTTTTAGATGTTCATGTTTTTGAATGTTTTATTTAAATAATATAAAAAATAAAATATATATTGACATGGATTAAACAAAGATATATATTATTCTATGTTGCATAAACAAATTGGCAAAATAGAGATGGAAGATAAAAATATGGTCAAAGTAATAAGAGTCTATGGTGAATGCCTA

**>G1PW494e_Borrelia afzelii_5S-23S ribosomal RNA intergenic spacer, partial sequence_Ixodes persulcatus_adult female_cloth-dragging_ytterstlandet**

GAGTTCGCGGGAGAGTAAGTTATTGCCAGGGTTTTTATTTTATACTTTAAACCTTGAATTTATTTTTTAAATGTTTATATTATTTGAATAAAACATTCAAATAATATAAAAAATAATATATATATTGACATGGATTAAACAAAGATATATATTATTTTATGTTGTATAAACAAATTGGCAAAATAGAGATGGAAGATAAAAATATGGTCAAAGTAATAAGAGTCTA

**>G2PW375a_Borrelia afzelii_5S-23S ribosomal RNA intergenic spacer, partial sequence_Ixodes persulcatus_adult male_cloth-dragging_ytterstlandet**

TTATACTTTAAACCTTGAATTTATTTTTTAAATGTTTATATTATTTGAATAAAACATTCAAATAATATAAAAAATAATATATATATTGACATGGATTAAACAAAGATATATATTATTTTATGTTGTATAAACAAATTGGCAAAATAGAGATGGAAGATAAAAATATGGTCAAAGTAATAAGAGTCTATGGTGAATGCCTA

**>G3PW375c_Borrelia garinii_5S-23S ribosomal RNA intergenic spacer, partial sequence_Ixodes persulcatus_adult male_cloth-dragging_ytterstlandet**

TTTTATTTTATACTTTAAACATTGATTTTATTTTTTATGTTTTTAGATGTTCATGTTTTTGAATGTTTTATTTAAATAATATAAAAAATAAAATATATATTGACATGGATTAAACAAAGATATATATTATTCTATGTTGCATAAGCAAATTGGCAAAATAGAGATGGAAGATAAAAATATGGTCAAAGTAATAAGAGTCTATGGTGAATGCCTA

**>G4PW495a_UNTYPEABLE_5S-23S ribosomal RNA intergenic spacer, partial sequence_Ixodes persulcatus_adult female_cloth-dragging_ytterstlandet**

No sequences obtained

**>G5PW495c_Borrelia garinii_5S-23S ribosomal RNA intergenic spacer, partial sequence_Ixodes persulcatus_adult female_cloth-dragging_ytterstlandet**

TTTATACTTTAAACATTGATTTTATTTTTTATGTTTTTAGATGTTCATGTTTTTGAATGTTTTATTTAAATAATATAAAAAATAAAATATATATTGACATGGATTAAACAAAGATATATATTATTCTATGTTGCATAAGCAAATTGGCAAAATAGAGATGGAAGATAAAAATATGGTCAAAGTAATAAGAGTCTATGGTGAATGCCTA

**>G6PW495d_Borrelia afzelii_5S-23S ribosomal RNA intergenic spacer, partial sequence_Ixodes persulcatus_adult female_cloth-dragging_ytterstlandet**

GTTATTGCCAGGGTTTTTATTTTATACTTTAAACCTTGAATTTATTTTTTAAATGTTTATATTATTTGAATAAAACATTCAAATAATATAAAAAATAATATATATATTGACATGGATTAAACAAAGATATATATTATTCTATGTTGTATGAACAAATTGGCAAAATAGAGATGGAAGATAAAAATATGGTCAAAGTAATAAGAGTCTATGGTGAATGCCTA

**>G7PW495e_Borrelia afzelii_5S-23S ribosomal RNA intergenic spacer, partial sequence_Ixodes persulcatus_adult female_cloth-dragging_ytterstlandet**

CGCGGGAGAGTAAGTTATTGCCAGGGTTTTTATTTTATACTTTAAACCTTGAATTTATTTTTTAAATGTTTATATTATTTGAATAAAACATTCAAATAATATAAAAAATAATATATATATTGACATGGATTAAACAAAGATATATATTATTCTATGTTGTATGAACAAATTGGCAAAATAGAGATGGAAGATAAAAATATGGTCAAAGTAATAAGAGTCTATGGTGAATGCCTA

**>G8PW495f_Borrelia garinii_5S-23S ribosomal RNA intergenic spacer, partial sequence_Ixodes persulcatus_adult female_cloth-dragging_ytterstlandet**

TATTTTATACTTTAAACATTGATTTTATTTTTTATGTTTTTAGATGTTCATGTTTTTGAATGTTTTATTTAAATAATATAAAAAATAAAATATATATTGACATGGATTAAACAAAGATATATATTATTCTATGTTGCATAAGCAAATTGGCAAAATAGAGATGGAAGATAAAAATATGGTCAAAGTAATAAGAGTCTATGGTGAATGCCTA

**>G9PW495h_UNTYPEABLE_5S-23S ribosomal RNA intergenic spacer, partial sequence_Ixodes persulcatus_adult female_cloth-dragging_ytterstlandet**

No sequences obtained

**>G10PW495i_UNTYPEABLE_5S-23S ribosomal RNA intergenic spacer, partial sequence_Ixodes persulcatus_adult female_cloth-dragging_ytterstlandet**

No sequences obtained

**>G11PW495j_Borrelia afzelii_5S-23S ribosomal RNA intergenic spacer, partial sequence_Ixodes persulcatus_adult female_cloth-dragging_ytterstlandet**

AAACCTTGAATTTATTTTTTAAATGTTTATATTATTTGAATAAAACATTCAAATAATATAAAAAATAATATATATATTGACATGGATTAAACAAAGATATATATTATTCTATGTTGTATGAACAAATTGGCAAAATAGAGATGGAAGATAAAAATATGGTCAAAGTAATAAGAGTCT

**>G12PW496a_Borrelia afzelii_5S-23S ribosomal RNA intergenic spacer, partial sequence_Ixodes persulcatus_adult male_cloth-dragging_ytterstlandet**

TTTTATACTTTAAACCTTGAATTTATTTTTTAAATGTTTATATTATTTGAATAAAACATTCAAATAATATAAAAAATAATATATATATTGACATGGATTAAACAAAGATATATATTATTCTATGTTGTATAAACAAATTGGCAAAATAGAGATGGAAGATAAAAATATGGTCAAAGTAATAAGAGTCTATGGTGAAT

**>H1PW496c_Borrelia afzelii_5S-23S ribosomal RNA intergenic spacer, partial sequence_Ixodes persulcatus_adult male_cloth-dragging_ytterstlandet**

GAGTTCGCGGGAGAGTAAGTTATTGCCAGGGTTTTTATTTTATACTTTAAACCTTGAATTTATTTTTTAAATGTTTATATTATTTGAATAAAACATTCAAATAATATAAAAAATAATATATATATTGACATGGATTAAACAAAGATATATATTATTCTATGTTGTATGAACAAATTGGCAAAATAGAGATGGAAGATAAAAAT

**>H2PW496d_Borrelia garinii_5S-23S ribosomal RNA intergenic spacer, partial sequence_Ixodes persulcatus_adult male_cloth-dragging_ytterstlandet**

TTATTTTATACTTTAAACATTGATTTTATTTTTTATGTTTTTAGATGTTCATGTTTTTGAATGTTTTATTTAAATAATATAAAAAATAAAATATATATTGACATGGATTAAACAAAGATATATATTATTCTATGTTGCATAAGCAAATTGGCAAAATAGAGATGGAAGATAAAAATATGGTCAAAGTAATAAGAGTCT

**>H3PW496e_Borrelia garinii_5S-23S ribosomal RNA intergenic spacer, partial sequence_Ixodes persulcatus_adult male_cloth-dragging_ytterstlandet**

GGTTTTTATTTTATACTTTAAACATTGATTTTATTTTTTATGTTTTTAGATGTTCATGTTTTTGAATGTTTTATTTAAATAATATAAAAAATAAAATATATATTGACATGGATTAAACAAAGATATATATTATTCTATGTTGCATAAACAAATTGGCAAAATAGAGATGGAAGATAAAAATATGGTCAAAGTAATAAGAGTCTT

**>H4PW496f_UNTYPEABLE_5S-23S ribosomal RNA intergenic spacer, partial sequence_Ixodes persulcatus_adult male_cloth-dragging_ytterstlandet**

No sequences obtained

**>H5PW496h_Borrelia afzelii_5S-23S ribosomal RNA intergenic spacer, partial sequence_Ixodes persulcatus_adult male_cloth-dragging_ytterstlandet**

GAGTTCGCGGGAGAGTAAGTTATTGCCAGGGTTTTTATTTTATACTTTAAACCTTGAATTTATTTTTTAAATGTTTATATTATTTGAATAAAACATTCAAATAATATAAAAAATAATATATATATTGACATGGATTAAACAAAGATATATATTATTTATGTTGTATGAACAAATTGGCAAAATAGAGATGGAAGATAAAAATATGGTCAAAGTAATAAGAGTCTATGGTGAATGCCTA

**>H6PW496j_Borrelia afzelii_5S-23S ribosomal RNA intergenic spacer, partial sequence_Ixodes persulcatus_adult male_cloth-dragging_ytterstlandet**

GAGTTCGCGGGAGAGTAAGTTATTGCCAGGGTTTTTATTTTATACTTTAAACCTTGAATTTATTTTTTAAATGTTTATATTATTTGAATAAAACATTCAAATAATATAAAAAATAATATATATATTGACATGGATTAAACAAAGATATATATTATTCTATGTTGTATGAACAAATTGGCAAAATAGAGATGGAAGATAAAAATATGGTCAAAGTAATAAGAGTCT

**>H7PW498a_Borrelia garinii_5S-23S ribosomal RNA intergenic spacer, partial sequence_Ixodes persulcatus_adult male_cloth-dragging_ytterstlandet**

GTTTTTATTTTATACTTTAAACATTGATTTTATTTTTTATGTTTTTAGATGTTCATGTTTTTGAATGTTTTATTTAAATAATATAAAAAATAAAATATATATTGACATGGATTAAACAAAGATATATATTATTCTATGTTGCATAAACAAATTGGCAAAATAGAGATGGAAGATAAAAATATGGTCAAAGTAATAAGAGTCTATGGTGAATGCCTA

**>H8PW498b_Borrelia afzelii_5S-23S ribosomal RNA intergenic spacer, partial sequence_Ixodes persulcatus_adult male_cloth-dragging_ytterstlandet**

AGTTCGCGGGAGAGTAAGTTATTGCCAGGGTTTTTATTTTATACTTTAAACCTTGAATTTATTTTTTAAATGTTTATATTATTTGAATAAAACATTCAAATAATATAAAAAATAATATATATATTGACATGGATTAAACAAAGATATATATTATTTTATGTTGTATAAACAAATTGGCAAAATAGAGATGGAAGATAAAAATATGGTCAAAGTAATAAGAGTCT

**>H9PW498c_Borrelia afzelii_5S-23S ribosomal RNA intergenic spacer, partial sequence_Ixodes persulcatus_adult male_cloth-dragging_ytterstlandet**

GAGTTCGCGGGAGAGTAAGTTATTGCCAGGGTTTTTATTTTATACTTTAAACCTTGAATTTATTTTTTAAATGTTTATATTATTTGAATAAAACATTCAAATAATATAAAAAATAATATATATATTGACATGGATTAAACAAAGATATATATTATTCTATGTTGTATGAACAAATTGGCAAAATAGAGATGGAAGATAAAAATATGGTCAAAGTAATAAGAGTCTATGGTGAATGCCTA

**>H10PW498d_Borrelia afzelii_5S-23S ribosomal RNA intergenic spacer, partial sequence_Ixodes persulcatus_adult male_cloth-dragging_ytterstlandet**

GAGTTCGCGGGAGAGTAAGTTATTGCCAGGGTTTTTATTTTATACTTTAAACCTTGAATTTATTTTTTAAATGTTTATATTATTTGAATAAAACATTCAAATAATATAAAAAATAATATATATATTGACATGGATTAAACAAAGATATATATTATTCTATGTTGTATGAACAAATTGGCAAAATAGAGATGGAAGATAAAAATATGGTCAAAGTAATAAGAGTCTA

**>H11PW498e_Borrelia afzelii_5S-23S ribosomal RNA intergenic spacer, partial sequence_Ixodes persulcatus_adult male_cloth-dragging_ytterstlandet**

GAGTTCGCGGGAGAGTAAGTTATTGCCAGGGTTTTTATTTTATACTTTAAACCTTGAATTTATTTTTTAAATGTTTATATTATTTGAATAAAACATTCAAATAATATAAAAAATAATATATATATTGACATGGATTAAACAAAGATATATATTATTTTATGTTGTATAAACAAATTGGCAAAATAGAGATGGAAGATAAAAATATGGTCAAAGTAATAAGAGTCTATGGTGAATGCCTA

**>A1PW498f_Borrelia afzelii_5S-23S ribosomal RNA intergenic spacer, partial sequence_Ixodes persulcatus_adult male_cloth-dragging_ytterstlandet**

GAGTTCGCGGGAGAGTAAGTTATTGCCAGGGTTTTTATTTTATACTTTAAACCTTGAATTTATTTTTTAAATGTTTATATTATTTGAATAAAACATTCAAATAATATAAAAAATAATATATATATTGACATGGATTAAACAAAGATATATATTATTCTATGTTGTATAAACAAATTGGCAAAATAGAGA TGGAAGATAAAAATATGGTC

**>A2PW498g_Borrelia afzelii_5S-23S ribosomal RNA intergenic spacer, partial sequence_Ixodes persulcatus_adult male_cloth-dragging_ytterstlandet**

TTTATACTTTAAACCTTGAATTTATTTTTTAAATGTTTATATTATTTGAATAAAACATTCAAATAATATAAAAAATAATATATATATTGACATGGATTAAACAAAGATATATATTATTTTATGTTGTATAAACAAATTGGCAAAATAGAGATGGAAGATAAAAATATGGTCAAAGTAATAAGAGTCTATGGTGAATGCCTA

**>A3PW498h_Borrelia afzelii_5S-23S ribosomal RNA intergenic spacer, partial sequence_Ixodes persulcatus_adult male_cloth-dragging_ytterstlandet**

TTATACTTTAAACCTTGAATTTATTTTTTAAATGTTTATATTATTTGAATAAAACATTCAAATAATATAAAAAATAATATATATATTGACATGGATTAAACAAAGATATATATTATTCTATGTTGTATGAACAAATTGGCAAAATAGAGATGGAAGATAAAAATATGGTCAAAGTAATAAGAGTCTATGGTGAATGCCTA

**>A4PW498j_Borrelia afzelii_5S-23S ribosomal RNA intergenic spacer, partial sequence_Ixodes persulcatus_adult male_cloth-dragging_ytterstlandet**

ACTTTAAACCTTGAATTTATTTTTTAAATGTTTATATTATTTGAATAAAACATTCAAATAATATAAAAAATAATATATATATTGACATGGATTAAACAAAGATATATATTATTCTATGTTGTATGAACAAATTGGCAAAATAGAGATGGAAGATAAAAATATGGTCAAAGTAATAAAGAGTCTATGGTGAATGCCTA

**>A5PW499a_Borrelia afzelii_5S-23S ribosomal RNA intergenic spacer, partial sequence_Ixodes persulcatus_adult male_cloth-dragging_ytterstlandet**

GAGTTCGCGGGAGAGTAAGTTATTGCCAGGGTTTTTATTTTATACTTTAAACCTTGAATTTATTTTTTAAATGTTTATATTATTTGAATAAAACATTCAAATAATATAAAAAATAATATATATATTGACATGGATTAAACAAAGATATATATTATTTTATGTTGTATAAACAAATTGGCAAAATAGAGATGGAAGATAAAAATATGGTCAAAGTAATAAGAGTCTATGGTGAATGCCTA

**>A6PW499b_ Borrelia afzelii_5S-23S ribosomal RNA intergenic spacer, partial sequence_Ixodes persulcatus_adult female_cloth-dragging_ytterstlandet**

GAGTTCGCGGGAGAGTAAGTTATTGCCAGGGTTTTTATTTTATACTTTAAACCTTGAATTTATTTTTTAAATGTTTATATTATTTGAATAAAACATTCAAATAATATAAAAAATAATATATATATTGACATGGATTAAACAAAGATATATATTATTCTATGTTGTATGAACAAATTGGCAAAATAGAGATGGAAGATAAAAATA

**>A7PW499c_Borrelia garinii_5S-23S ribosomal RNA intergenic spacer, partial sequence_Ixodes persulcatus_adult female_cloth-dragging_ytterstlandet**

GGTTTTTATTTTATACTTTAAACATTGATTTTATTTTTTATGTTTTTAGATGTTCATGTTTTTGAATGTTTTATTTAAATAATATAAAAAATAAAATATATATTGACATGGATTAAACAAAGATATATATTATTCTATGTTGCATAAAC AAATTGGCAAAATAGAGATGGAAGATAAAAATATGGTCAAAGTAATAAGAGTCTATGGTGAATGCCTA

**>A8PW499d_Borrelia garinii_5S-23S ribosomal RNA intergenic spacer, partial sequence_Ixodes persulcatus_adult female_cloth-dragging_ytterstlandet**

TTTATACTTTAAACATTGATTTTATTTTTTATGTTTTTAGATGTTCATGTTTTTGAATGTTTTATTTAAATAATATAAAAAATAAAATATATATTGACATGGATTAAACAAAGATATATATTATTCTATGTTGCATAAGCAAATTGGCAAAATAGAGATGGAAGATAAAAATATGGTCAAAGTAATAAGAGTC

**>A9PW499g_Borrelia garinii_5S-23S ribosomal RNA intergenic spacer, partial sequence_Ixodes persulcatus_adult female_cloth-dragging_ytterstlandet**

GGTTTTTATTTTATACTTTAAACATTGATTTTATTTTTTATGTTTTTAGATGTTCATGTTTTTGAATGTTTTATTTAAATAATATAAAAAATAAAATATATATTGACATGGATTAAACAAAGATATATATTATTCTATGTTGCATAAGCAAATTGGCAAAATAGAGATGGAAGATAAAAATATGGTCAAAGTAATAAGAGTC

**>A10PW499i_Borrelia afzelii_5S-23S ribosomal RNA intergenic spacer, partial sequence_Ixodes persulcatus_adult female_cloth-dragging_ytterstlandet**

GAGTTCGCGGGAGAGTAAGTTATTGCCAGGGTTTTTATTTTATACTTTAAACCTTGAATTTATTTTTTAAATGTTTATATTATTTGAATAAAACATTCAAATAATATAAAAAATAATATATATATTGACATGGATTAAACAAAGATATATATTATTTTATGTTGTATAAACAAATTGGCAAAATAGAGATGAAGATAAAAATATGGTCAAAGTAATAAGAGTCTATGGTGAATGCCTA

**>A11PW499j_UNTYPEABLE_5S-23S ribosomal RNA intergenic spacer, partial sequence_Ixodes persulcatus_adult female_cloth-dragging_ytterstlandet**

No sequences obtained

**>A12PW369b_Borrelia garinii_5S-23S ribosomal RNA intergenic spacer, partial sequence_Ixodes persulcatus_adult female_cloth-dragging_ytterstlandet**

GGTTTTTATTTTATACTTTAAACATTGATTTTATTTTTTATGTTTTTAGATGTTCATGTTTTTGAATGTTTTATTTAAATAATATAAAAAATAAAATATATATTGACATGGATTAAACAAAGATATATATTATTCTATGTTGCATAAACAAATTGGCAAAATAGAGATGGAAGATAAAAATATGGTCAAAGTAATAAGAGTCTATGGTGAATGCCTA

**>B1PW369c_Borrelia afzelii_5S-23S ribosomal RNA intergenic spacer, partial sequence_Ixodes persulcatus_adult female_cloth-dragging_ytterstlandet**

GAGTTCGCGGGAGAGTAAGTTATTGCCAGGGTTTTTATTTTATACTTTAAACCTTGAATTTATTTTTTAAATGTTTATATTATTTGAATAAAACATTCAAATAATATAAAAAATAATATATATATTGACATGGATTAAACAAAGATATATATTATTCTATGTTGTATGAACAAATTGGCAAAATAGAGATGGAAGATAAAAATATGGTCAAAGTAATAAGAGTCTATGGTGAATGCCTA

**>B2PW369e_UNTYPEABLE_5S-23S ribosomal RNA intergenic spacer, partial sequence_Ixodes persulcatus_adult female_cloth-dragging_ytterstlandet**

No sequences obtained

**>B3PW369h_Borrelia garinii_5S-23S ribosomal RNA intergenic spacer, partial sequence_Ixodes persulcatus_adult female_cloth-dragging_ytterstlandet**

GGTTTTTATTTTATACTTTAAACATTGATTTTATTTTTTATGTTTTTAGATGTTCATGTTTTTGAATGTTTTATTTAAATAATATAAAAAATAAAATATATATTGACATGGATTAAACAAAGATATATATTATTCTATGTTGCATAAGCAAATTGGCAAAATAGAGATGGAAGATAAAAATATGGTCAAAGTAATAAGAGTCTATGG

**>B4PW369j_Borrelia afzelii_5S-23S ribosomal RNA intergenic spacer, partial sequence_Ixodes persulcatus_adult female_cloth-dragging_ytterstlandet**

GAGTTCGCGGGAGAGTAAGTTATTGCCAGGGTTTTTATTTTATACTTTAAACCTTGAATTTATTTTTTAAATGTTTATATTATTTGAATAAAACATTCAAATAATATAAAAAATAATATATATATTGACATGGATTAAACAAAGATATATATTATTCTATGTTGTATGAACAAATTGGCAAAATAGAGATGGAAGATAAAAATATGGTCAAAGTAATAAGAGTCTATGGT

**>B5PW370a_Borrelia afzelii_5S-23S ribosomal RNA intergenic spacer, partial sequence_Ixodes persulcatus_adult female_cloth-dragging_ytterstlandet**

GAGTTCGCGGGAGAGTAAGTTATTGCCAGGGTTTTTATTTTATACTTTAAACCTTGAATTTATTTTTTAAATGTTTATATTATTTGAATAAAACATTCAAATAATATAAAAAATAATATATATATTGACATGGATTAAACAAAGATATATATTATTCTATGTTGTATGAACAAATTGGCAAAATAGAGATGGAAGATAAAAATATGGTCAAAGTAATAAGAGTCTATGGTGAATGCCTA

**>B6PW370c_Borrelia afzelii_5S-23S ribosomal RNA intergenic spacer, partial sequence_Ixodes persulcatus_adult female_cloth-dragging_ytterstlandet**

GAGTTCGCGGGAGAGTAAGTTATTGCCAGGGTTTTTATTTTATACTTTAAACCTTGAATTTATTTTTTAAATGTTTATATTATTTGAATAAAACATTCAAATAATATAAAAAATAATATATATATTGACATGGATTAAACAAAGATATATATTATTTTATGTTGTATGAACAAATTGGCAAAATAGAGATGGAAGATAAAAATATGGTCAAAGTAATAAGAGTCTATGGTGAATGCCTA

**>B7PW370d_Borrelia afzelii_5S-23S ribosomal RNA intergenic spacer, partial sequence_Ixodes persulcatus_adult female_cloth-dragging_ytterstlandet**

GAATAAAACATTCAAATAATATAAAAAATAATATATATATTGACATGGATTAAACAAAGATATATATTATTCTATGTTGTATGAACAAATTGGCAAAATAGAGATGGAAGATAAAAATATGGTCAAAGTAATAAGAGTCTATGGTGAATGCCTAGGA

**>B8PW370g_Borrelia garinii_5S-23S ribosomal RNA intergenic spacer, partial sequence_Ixodes persulcatus_adult female_cloth-dragging_ytterstlandet**

GGTTTTTATTTTATACTTTAAACATTGATTTTATTTTTTATGTTTTTAGATGTTCATGTTTTTGAATGTTTTATTTAAATAATATAAAAAATAAAATATATATTGACATGGATTAAACAAAGATATATATTATTCTATGTTGCATAAACAAATTGGCAAAATAGAGATGGAAGATAAAAATATGGTCAAAGTAATAAGAGTCTATGGTGAATGCCTA

**>B9PW370h_Borrelia afzelii_5S-23S ribosomal RNA intergenic spacer, partial sequence_Ixodes persulcatus_adult female_cloth-dragging_ytterstlandet**

GAGTTCGCGGGAGAGTAAGTTATTGCCAGGGTTTTTATTTTATACTTTAAACCTTGAATTTATTTTTTAAATGTTTATATTATTTGAATAAAACATTCAAATAATATAAAAAATAATATATATATTGACATGGATTAAACAAAGATATATATTATTTTATGTTGTATAAACAAATTGGCAAAATAGAGATGGAAGATAAAAATATGGTCAAAGTAATAAGAGTCT

**>B10PW370i_Borrelia afzelii_5S-23S ribosomal RNA intergenic spacer, partial sequence_Ixodes persulcatus_adult female_cloth-dragging_ytterstlandet**

GAGTAAGTTATTGCCAGGGTTTTTATTTTATACTTTAAACCTTGAATTTATTTTTTAAATGTTTATATTATTTGAATAAAACATTCAAATAATATAAAAAATAATATATATATTGACATGGATTAAACAAAGATATATATTATTTTATGTTGTATGAACAAATTGGCAAAATAGAGATGGAAGATAAAAATATGGTCAAAGTAATAAGAGTCTATGGTGAATGCCTA

**>B11PW370j_Borrelia afzelii_5S-23S ribosomal RNA intergenic spacer, partial sequence_Ixodes persulcatus_adult female_cloth-dragging_ytterstlandet**

CTTTAAACCTTGAATTTATTTTTTAAATGTTTATATTATTTGAATAAAACATTCAAATAATATAAAAAATAATATATATATTGACATGGATTAAACAAAGATATATATTATTTTATGTTGTATAAACAAATTGGCAAAATAGAGATGGAAGATAAAAATATGGTCAAAGTAATAAGAGTCT

**>B12PW374a_Borrelia afzelii_5S-23S ribosomal RNA intergenic spacer, partial sequence_Ixodes persulcatus_adult female_cloth-dragging_ytterstlandet**

GAGTTCGCGGGAGAGTAAGTTATTGCCAGGGTTTTTATTTTATACTTTAAACCTTGAATTTATTTTTTAAATGTTTATATTATTTGAATAAAACATTCAAATAATATAAAAAATAATATATATATTGACATGGATTAAACAAAGATATATATTATTCTATGTTGTATGAACAAATTGGCAAAATAGAGATGGAAGATAAAAATATGGTCAAAGTAATAAGAGTCTATGGTG

**>C1PW374c_Borrelia afzelii_5S-23S ribosomal RNA intergenic spacer, partial sequence_Ixodes persulcatus_adult female_cloth-dragging_ytterstlandet**

GAGTTCGCGGGAGAGTAAGTTATTGCCAGGGTTTTTATTTTATACTTTAAACCTTGAATTTATTTTTTAAATGTTTATATTATTTGAATAAAACATTCAAATAATATAAAAAATAATATATATATTGACATGGATTAAACAAAGATATATATTATTTTATGTTGTATAAACAAATTGGCAAAATAGAGATGGAAGATAAAAATATGGTCAAAGTAATAAGAGTCTA

**>C2PW347d_UNTYPEABLE_5S-23S ribosomal RNA intergenic spacer, partial sequence_Ixodes persulcatus_adult female_cloth-dragging_ytterstlandet**

No sequences obtained

**>C3PW371a_Borrelia afzelii_5S-23S ribosomal RNA intergenic spacer, partial sequence_Ixodes persulcatus_adult male_cloth-dragging_ytterstlandet**

GAGTTCGCGGGAGAGTAAGTTATTGCCAGGGTTTTTATTTTATACTTTAAACCTTGAATTTATTTTTTAAATGTTTATATTATTTGAATAAAACATTCAAATAATATAAAAAATAATATATATATTGACATGGATTAAACAAAGATATATATTATTCTATGTTGTATGAACAAATTGGCAAAATAGAGATGGAAGATAAAAATATGGTCAAAGTAATAAGAGT

**>C4PW371b_Borrelia garinii_5S-23S ribosomal RNA intergenic spacer, partial sequence_Ixodes persulcatus_adult male_cloth-dragging_ytterstlandet**

GTTATTGAAAGAGGTTTTTATTTTATACTTTAAACATTGATTTTATTTTTTATGTTTTTAGATGTTCATGTTTTTGAATGTTTTATTTAAATAATATAAAAAATAAAATATATATTGACATGGATTAAACAAAGATATATATTATTCTATGTTGCATAAGCAAATTGGCAAAATAGAGATGGAAGATAAAAATATGGTCAAAGTAATAAGAG

**>C5PW371c_Borrelia garinii_5S-23S ribosomal RNA intergenic spacer, partial sequence_Ixodes persulcatus_adult male_cloth-dragging_ytterstlandet**

AGAGGTTTTTATTTTATACTTTAAACATTGATTTTATTTTTTATGTTTTTAGATGTTCATGTTTTTGAATGTTTTATTTAAATAATATAAAAAATAAAATATATATTGACATGGATTAAACAAAGATATATATTATTCTATGTTGCATAAGCAAATTGGCAAAATAGAGATGGAAGATAAAAATATGGTCAAAGTAATAAGAGTC

**>C6PW371d_Borrelia afzelii_5S-23S ribosomal RNA intergenic spacer, partial sequence_Ixodes persulcatus_adult male_cloth-dragging_ytterstlandet**

GAGTTCGCGGGAGAGTAAGTTATTGCCAGGGTTTTTATTTTATACTTTAAACCTTGAATTTATTTTTTAAATGTTTATATTATTTGAATAAAACATTCAAATAATATAAAAAATAATATATATATTGACATGGATTAAACAAAGATATATATTATTCTATGTTGTATGAACAAATTGGCAAAATAGAGATGGAAGATAAAAATATGGTCAAAGTAATAAGAGTCTA

**>C7PW371e_Borrelia garinii_5S-23S ribosomal RNA intergenic spacer, partial sequence_Ixodes persulcatus_adult male_cloth-dragging_ytterstlandet**

GGTTTTTATTTTATACTTTAAACATTGATTTTATTTTTTATGTTTTTAGATGTTCATGTTTTTGAATGTTTTATTTAAATAATATAAAAAATAAAATATATATTGACATGGATTAAACAAAGATATATATTATTCTATGTTGCATAAGCAAATTGGCAAAATAGAGATGGAAGATAAAAATATGGTCAAAGTAATAAGAG

**>C8PW371f_ Borrelia afzelii_5S-23S ribosomal RNA intergenic spacer, partial sequence_Ixodes persulcatus_adult male_cloth-dragging_ytterstlandet**

GAGTTCGCGGGAGAGTAAGTTATTGCCAGGGTTTTTATTTTATACTTTAAACCTTGAATTTATTTTTTAAATGTTTATATTATTTGAATAAAACATTCAAATAATATAAAAAATAATATATATATTGACATGGATTAAACAAAGATATATATTATTTTATGTTGTATGAACAAATTGGCAAAATAGAGATGGAAGATAAAAATATGGTCAAAGTAATAAGAGTCTATGGTGAATGCCTA

**>C9PW371j_Borrelia garinii_5S-23S ribosomal RNA intergenic spacer, partial sequence_Ixodes persulcatus_adult male_cloth-dragging_ytterstlandet**

GGTTTTTATTTTATACTTTAAACATTGATTTTATTTTTTATGTTTTTAGATGTTCATGTTTTTGAATGTTTTATTTAAATAATATAAAAAATAAAATATATATTGACATGGATTAAACAAAGATATATATTATTCTATGTTGCATAAGCAAATTGGCAAAATAGAGATGGAAGATAAAAATATGGTCAAAGTAATAAGAGTCTATGGTGAATGCCTA

**>C10PW372a_Borrelia afzelii_5S-23S ribosomal RNA intergenic spacer, partial sequence_Ixodes persulcatus_adult male_cloth-dragging_ytterstlandet**

TATTTTATACTTTAAACCTTGAATTTATTTTTTAAATGTTTATATTATTTGAATAAAACATTCAAATAATATAAAAAATAATATATATATTGACATGGATTAAACAAAGATATATATTATTCTATGTTGTATGAACAAATTGGCAAAATAGAGATGGAAGATAAAAATATGGTCAAAGTAATAAGAGTCTATGGTGAATGCCT

**>C11PW372b_Borrelia afzelii_5S-23S ribosomal RNA intergenic spacer, partial sequence_Ixodes persulcatus_adult male_cloth-dragging_ytterstlandet**

GAGTTCGCGGGAGAGTAAGTTATTGCCAGGGTTTTTATTTTATACTTTAAACCTTGAATTTATTTTTTAAATGTTTATATTATTTGAATAAAACATTCAAATAATATAAAAAATAATATATATATTGACATGGATTAAACAAAGATATATATTATTTTATGTTGTATAAACAAATTGGCAAAATAGAGATGGAAGATAAAAATATGGTCAAAGTAATAAGAGTCTATGGTGAATGCCTA

**>C12PW372e_UNTYPEABLE_5S-23S ribosomal RNA intergenic spacer, partial sequence_Ixodes persulcatus_adult male_cloth-dragging_ytterstlandet**

No sequences obtained

**>D1PW372f_Borrelia garinii_5S-23S ribosomal RNA intergenic spacer, partial sequence_Ixodes persulcatus_adult male_cloth-dragging_ytterstlandet**

TTTTTATTTTATACTTTAAACATTGATTTTATTTTTTATGTTTTTAGATGTTCATGTTTTTGAATGTTTTATTTAAATAATATAAAAAATAAAATATATATTGACATGGATTAAACAAAGATATATATTATTCTATGTTGCATAAGCAAATTGGCAAAATAGAGATGGAAGATAAAAATATGGTCAAAGTAATAAGAGTCT

**>D2PW372g_UNTYPEABLE_5S-23S ribosomal RNA intergenic spacer, partial sequence_Ixodes persulcatus_adult male_cloth-dragging_ytterstlandet**

No sequences obtained

**>D3PW372i_Borrelia garinii_5S-23S ribosomal RNA intergenic spacer, partial sequence_Ixodes persulcatus_adult male_cloth-dragging_ytterstlandet**

GGTTTTTATTTTATACTTTAAACATTGATTTTATTTTTTATGTTTTTAGATGTTCATGTTTTTGAATGTTTTATTTAAATAATATAAAAAATAAAATATATATTGACATGGATTAAACAAAGATATATATTATTCTATGTTGCATAAGCAAATTGGCAAAATAGAGATGGAAGATAAAAATATGGTCAAAGTAATAAGAGTCTATGGTGAATGCCTA

**>D4PW373a_Borrelia garinii_5S-23S ribosomal RNA intergenic spacer, partial sequence_Ixodes persulcatus_adult male_cloth-dragging_ytterstlandet**

GGTTTTTATTTTATACTTTAAACATTGATTTTATTTTTTATGTTTTTAGATGTTCATGTTTTTGAATGTTTTATTTAAATAATATAAAAAATAAAATATATATTGACATGGATTAAACAAAGATATATATTATTCTATGTTGCATAA ACAAATTGGCAAAATAGAGATGGAAGATAAAAATATGGTCAAAGTAATAAGAGTCT

**>D5PW373b_Borrelia valaisiana_5S-23S ribosomal RNA intergenic spacer, partial sequence_Ixodes persulcatus_adult male_cloth-dragging_ytterstlandet**

GAGTTCGCGGGAGAGTAAGTTATTGCCAGGGTTTTTATTTTGTAATTTAAACCTTAAATTTATTTTTTATATTTTTTTAATGTTCATGTTTTTGAATGTTTTATTCAAATAATGTAAAAAATAAAATAGATATTGACATGGATTGAACAAAAGATATATATTATTTTATGTTGCATAAACAAATTGGCAAAATAGAGATGGAAGATAAAAATATGGTCAAAG

**>D6PW373d_Borrelia garinii_5S-23S ribosomal RNA intergenic spacer, partial sequence_Ixodes persulcatus_adult male_cloth-dragging_ytterstlandet**

GGTTTTTATTTTATACTTTAAACATTGATTTTATTTTTTATGTTTTTAGATGTTCATGTTTTTGAATGTTTTATTTAAATAATATAAAAAATAAAATATATATTGACATGGATTAAACAAAGATATATATTATTCTATGTTGCATAAGCAAATTGGCAAAATAGAGATGGAAGATAAAAATATGGTCAAAGTAATAAGAGTCTATGGTGAATGCCTA

**>D7PW377b_Borrelia afzelii_5S-23S ribosomal RNA intergenic spacer, partial sequence_Ixodes persulcatus_adult male_cloth-dragging_ytterstlandet**

GAGTTCGCGGGAGAGTAAGTTATTGCCAGGGTTTTTATTTTATACTTTAAACCTTGAATTTATTTTTTAAATGTTTATATTATTTGAATAAAACATTCAAATAATATAAAAAATAATATATATATTGACATGGATTAAACAAAGATATATATTATTCTATGTTGTATGAACAAATTGGCAAAATAGAGATGGAAGATAAAAATATGGTCAAAGTAATAAGAGTCT

**>D8PW377e_UNTYPEABLE_5S-23S ribosomal RNA intergenic spacer, partial** **sequence_Ixodes persulcatus_adult male_cloth-dragging_ytterstlandet**

No sequences obtained

**>D9PW377f_Borrelia afzelii_5S-23S ribosomal RNA intergenic spacer, partial sequence_Ixodes persulcatus_adult male_cloth-dragging_ytterstlandet**

CTCTATTTTGCCAATTTGTTTATACAACATAAAATAATATATATCTTTGTTTAATCCATGTCAATATATATATTATTTTTTATATTATTTGAATGTTTTATTCAAATAATATAAACATTTAAAAAATAAATTCAAGGTTTAAAGTATAAAATAAAAACCCTGGCAATAACTTACTCTCCCGCGAACTC

**>D10PW377j_UNTYPEABLE_5S-23S ribosomal RNA intergenic spacer, partial sequence_Ixodes persulcatus_adult male_cloth-dragging_ytterstlandet**

No sequences obtained

**>D11PW378a_Borrelia afzelii_5S-23S ribosomal RNA intergenic spacer, partial sequence_Ixodes persulcatus_adult female_cloth-dragging_ytterstlandet**

GAGTAAGTTATTGCCAGGGTTTTTATTTTATACTTTAAACCTTGAATTTATTTTTTAAATGTTTATATTATTTGAATAAAACATTCAAATAATATAAAAAATAATATATATATTGACTGGATTAAACAAAGATATATATTATTCTATGTTGTATGAACAAATTGGCAAAATAGAGATGGAAGATAAAAATATGGTCAAAGTAATAAGAGTCTATGGTGAATGCCTA

**>D12PW378c_Borrelia afzelii_5S-23S ribosomal RNA intergenic spacer, partial sequence_Ixodes persulcatus_adult female_cloth-dragging_ytterstlandet**

GAGTTCGCGGGAGAGTAAGTTATTGCCAGGGTTTTTATTTTATACTTTAAACCTTGAATTTATTTTTTAAATGTTTATATTATTTGAATAAAACATTCAAATAATATAAAAAATAATATATATATTGACATGGATTAAACAAAGATATATATTATTCTATGTTGTATGAACAAATTGGCAAAATAGAGATGGAAGATAAAAATATGGTCAAAGTAATAAGAGTCT

**>E1PW378d_Borrelia afzelii_5S-23S ribosomal RNA intergenic spacer, partial sequence_Ixodes persulcatus_adult female_cloth-dragging_ytterstlandet**

CTTGAATTTATTTTTTAAATGTTTATATTATTTGAATAAAACATTCAAATAATATAAAAAATAATATATATATTGACATGGATTAAACAAAGATATATATTATTTTATGTTGTATGAACAAATTGGCAAAATAGAGATGGAAGATAAAAATATGGTCAAAGTAATAAGAGTCTATGGTGAATGCCTA

**>E2PW378e_Borrelia afzelii_5S-23S ribosomal RNA intergenic spacer, partial sequence_Ixodes persulcatus_adult female_cloth-dragging_ytterstlandet**

CCTTGAATTTATTTTTTAAATGTTTATATTATTTGAATAAAACATTCAAATAATATAAAAAATAATATATATATTGACATGGATTAAACAAAGATATATATTATTCTATGTTGTATGAACAAATTGGCAAAATAGAGATGGAAGATAAAAATATGGTCAAAGTAATAAGAGTCTATGGTGAATGCCTA

**>E3PW378f_UNTYPEABLE_5S-23S ribosomal RNA intergenic spacer, partial sequence_Ixodes persulcatus_adult female_cloth-dragging_ytterstlandet**

No sequences obtained

**>E4PW378g_UNTYPEABLE_5S-23S ribosomal RNA intergenic spacer, partial sequence_Ixodes persulcatus_adult female_cloth-dragging_ytterstlandet**

No sequences obtained

**>E5PW378h_Borrelia garinii_5S-23S ribosomal RNA intergenic spacer, partial sequence_Ixodes persulcatus_adult female_cloth-dragging_ytterstlandet**

TTATACTTTAAACATTGATTTTATTTTTTATGTTTTTAGATGTTCATGTTTTTGAATGTTTTATTTAAATAATATAAAAAATAAAATATATATTGACATGGATTAAACAAAGATATATATTATTCTATGTTGCATAAGCAAATTGGCAAAATAGAGATGGAAGATAAAAATATGGTCAAAGTAATAAGAGTCTATGGTGAATGCCTA

**>E6PW378i_Borrelia afzelii_5S-23S ribosomal RNA intergenic spacer, partial sequence_Ixodes persulcatus_adult female_cloth-dragging_ytterstlandet**

GAGTTCGCGGGAGAGTAAGTTATTGCCAGGGTTTTTATTTTATACTTTAAACCTTGAATTTATTTTTTAAATGTTTATATTATTTGAATAAAACATTCAAATAATATAAAAAATAATATATATATTGACATGGATTAAACAAAGATATATATTATTTTATGTTGTATAAACAAATTGGCAAAATAGAGATGGAAGATAAAAATATGGTCAAAGTAATAAGAGTCTATGGTGAATGCCTA

**>E7PW379b_Borrelia garinii_5S-23S ribosomal RNA intergenic spacer, partial sequence_Ixodes persulcatus_adult male_cloth-dragging_ytterstlandet**

GAATGTTTTATTTAAATAATATAAAAAATAAAATATATATTGACATGGATTAAACAAAGATATATATTATTCTATGTTGCATAAGCAAATTGGCAAAATAGAGATGGAAGATAAAAATATGGTCAAAGTAATAAGAGTCTATGGTGAATGCCTAGGA

**>E8PW379e_Borrelia garinii_5S-23S ribosomal RNA intergenic spacer, partial sequence_Ixodes persulcatus_adult male_cloth-dragging_ytterstlandet**

GATGTTCATGTTTTTGAATGTTTTATTTAAATAATATAAAAAATAAAATATATATTGACATGGATTAAACAAAGATATATATTATTCTATGTTGCATAAGCAAATTGGCAAAATAGAGATGGAAGATAAAAATATGGTCAAAGTAATAAGAGTCTATGGTGAATGCCTA

**>E9PW380a_Borrelia afzelii_5S-23S ribosomal RNA intergenic spacer, partial sequence_Ixodes persulcatus_adult male_removed from a dog_ytterstlandet**

GAGTTCGCGGGAGAGTAAGTTATTGCCAGGGTTTTTATTTTATACTTTAAACCTTGAATTTATTTTTTAAATGTTTATATTATTTGAATAAAACATTCAAATAATATAAAAAATAATATATATATTGACATGGATTAAACAAAGATATATATTATTCTATGTTGTATGAACAAATTGGCAAAATAGAGATGGAAGATAAAAATATGGTCAAAGTAATAAGAGTCTATGGTGAATGCCTA

**>E10PW380f_UNTYPEABLE_5S-23S ribosomal RNA intergenic spacer, partial sequence_Ixodes persulcatus_adult male_removed from a dog_ytterstlandet**

No sequences obtained

**>E11PW380h_UNTYPEABLE_5S-23S ribosomal RNA intergenic spacer, partial sequence_Ixodes persulcatus_adult male_removed from a dog_ytterstlandet**

No sequences obtained

**>E12PW381a_UNTYPEABLE_5S-23S ribosomal RNA intergenic spacer, partial sequence_Ixodes persulcatus_adult female_removed from a dog_ytterstlandet**

No sequences obtained

**>F1PW381c_Borrelia afzelii_5S-23S ribosomal RNA intergenic spacer, partial sequence_Ixodes persulcatus_adult female_removed from a dog_ytterstlandet**

ATTTTATACTTTAAACCTTGAATTTATTTTTTAAATGTTTATATTATTTGAATAAAACATTCAAATAATATAAAAAATAATATATATATTGACATGGATTAAACAAAGATATATATTATTCTATGTTGTATGAACAAATTGGCAAAATAGAGATGGAAGATAAAAATATGGTCAAAGTAATAAGAGTCTATGGTGAATGCCTA

**>Batch15PWR164b_Borrelia garinii_5S-23S ribosomal RNA intergenic spacer, partial sequence_Ixodes persulcatus_adult female_cloth-dragging_västra knivskär**

GGTTTTTATTTTATACTTTAAACATTGATTTTATTTTTTATGTTTTTAGATGTTCATGTTTTTGAATGTTTTATT CAAATAATATAAAAAATAAAATATATATTGACATGGATTAAACAAAGATATATATTATTCTATGTTGTATAAACAAATTGGCAAAATAGAGATGGAAGATAAAAATATGGTCAAAGTAATAAGAGTCT

**>Batch15PW853b_Borrelia garinii_5S-23S ribosomal RNA intergenic spacer, partial sequence_Ixodes persulcatus_nymph_cloth-dragging_ytterstlandet**

GGTTTTTATTTTATACTTTAAACATTGATTTTATTTTTTATGTTTTTAGATGTTCATGTTTTTGAATGTTTTATTTAAATAATATAAAAAATAAAATATATATTGACATGGATTAAACAAAGATATATATTATTCTATGTTGCATAAACAAATTGGCAAAATAGAGATGGAAGATAAAAATATGGTCAAAGTAATAAGAGTCTATGGTGAATGCCTA

**>PW480e_Rickettsia helvetica_ompB_Ixodes persulcatus_adult female_cloth-dragging_västra knivskär**

GGACTTAGCTACTTAAAGTCTTCTGACGAAAACTATAAAGAAACCGGTACAACAGTTGCAAACAAGCAAGTTAACAGCAAGTTTAGCGATAGAACTGATTTAATAGTAGGTGCTAAAGTAGCTGGCGGTACTATGAACATAACTGATCTTGCGGTATATCCGGAAGCTCACGCTTTTGTGGTTCACAAAGTAAACGGTAGATTATCTAAAACTCAGTCTGTGTTAGACGGACAAGTTACTCCGT

**>PW480e_Rickettsia helvetica_gltA_Ixodes persulcatus_adult female_cloth-dragging_västra knivskär**

CAAGGTACAGGAACACTTCTTGGCGGTGCCGGCGGTGCATTACTTGGTTCTCAATTTGGTAAAGGTAAAGGGCAACTTGTCGGAGTAGGTGTAGGTGCATTACTTGGAGCAGTTCTTGGCGGGCAAATCGGTGCAGGTATGGATGAGCAGGATAGAAGACTTGCAGAGCTTACCTCACAGAGAGCTTTAGAAGCAGCTCCTAGCGGTAGTAACGTAGAGTGGCGTAATCCGGATAACGGCAATTACGGTTACGTAACACCTAATAAAACTTATAGAAATAGCACTGGTCAATATTGCCGTGAGTACACTCAAACAGTTGTAATAG

**>PW950_Rickettsia_sp._ompB_Ixodes persulcatus_nymph_cloth-dragging_östra knivskär**

CCAATGGCAGGACTTAGCTACTTGAAGTCTTCTGACGAAAACTACAAAGAAACCGGTACAACAGTTGCAAACAAGCAAGTTAACAGCAAGTTTAGCGATAGAACCGATTTAATAGTAGGTGCTAAAGTAATGGGTAATACTATGAACTTTAGTGATCTTGCGGTATATCCGGAAGCTCATGCTTTTGTGGTTCACAAAGTAAACGGTAGATTATCTAAAATTCAGTCTCAGTTAGACGGACAAGTTACTCCGTGTATCAGCCAGCCT
